# Sulfonium‐Based Antimicrobial Block Copolymers: Influence of Hydrophobicity on Biological Activity and Antibiotic Synergy

**DOI:** 10.1002/marc.202500421

**Published:** 2025-09-30

**Authors:** Sidra Kanwal, Otto Staudhammer, Umer Bin Abdul Aziz, Elisa Quaas, Jörg Rademann, Daniel Klinger

**Affiliations:** ^1^ Freie Universität Berlin Institute of Pharmacy Berlin Germany; ^2^ Freie Universität Berlin Department of Biology, Chemistry, Pharmacy and Physics SupraFAB Berlin Germany

**Keywords:** antibiotics, antimicrobial polymers, block copolymers, ciprofloxacin, penicillin G, sulfonium cations, synergistic interactions

## Abstract

Antimicrobial polymers (AMP) are promising therapeutics to target bacterial pathogens. Sulfonium‐based AMPs offer a good balance between high antimicrobial activity and low cytotoxicity. Currently, sulfonium groups are mostly incorporated into random copolymers that combine these cations with hydrophobic and neutral hydrophilic groups. In contrast, sulfonium‐based block copolymers (BCP), that structurally separate these functionalities, are less explored with structure‐property relations missing. Addressing this gap, we investigated BCPs that combine the active sulfonium‐based block with a neutral hydrophilic poly(polyethylene glycol methacrylate) (PPEGMA) block to improve cytocompatibility. The sulfonium cations contain varying ratios of two different hydrophobic side groups, i.e., benzyl (*bz*) and methyl (*me*) groups. By changing the *bz*:*me* ratio, we adjusted the overall polymer hydrophobicity. BCPs with *bz* contents above 30 mol% showed the highest activity against *E. coli* and *S. aureus* whereas those with *bz* contents ≤ 30 mol% exhibited the best cell viability. Thus, a *bz* content of 30 mol% offers optimal balance between antimicrobial activity and cytotoxicity. Combining these polymers with small molecule antibiotics penicillin G and ciprofloxacin resulted in synergistic effects, reducing the required concentrations of both polymer and antibiotic. These findings position sulfonium‐based BCPs as a promising platform to boost the efficacy of conventional antibiotics.

## Graphical abstract

Sulfonium‐based antimicrobial block copolymers combine an active cationic hydrophobic block with a neutral hydrophilic block to improve cytocompatibility. The hydrophobicity of the sulfonium‐based block controls the activity against *S. aureus* and *E coli*. Combining these polymers with small molecule antibiotics penicillin G and ciprofloxacin gives synergistic effects, reducing the required concentrations of both polymer and antibiotic.

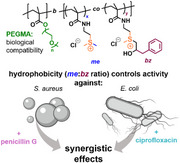

## 1 Introduction

Antimicrobial resistance (AMR) has emerged as critical threat to global human health [1, 2, 3, 4, 5]. As pathogens evolve resistance to conventional antibiotics [6, 7], such small‐molecule drugs are increasingly defeated by enzymatic degradation, efflux pumps, and target modifications [8, 9]. As a result, innovative therapeutic approaches are needed to circumvent such potential failure pathways. In this context, antimicrobial polymers (AMPs) have emerged as promising alternatives that mimic the cationic and amphiphilic structure of natural host‐defense peptides (HDPs) [10, 11, 12, 13, 14, 15]. These polymers disrupt bacterial membranes through a combination of electrostatic and hydrophobic interactions thereby resulting in lysis and bacterial death [16]. Compared to conventional antibiotics, such a non‐specific mode of action hinders the development of resistance mechanisms, thus suggesting robust therapeutic activity [11, 17].

During the development of cationic antimicrobial polymers, potential cytotoxicity needs to be addressed. Such unwanted side effects are the result of electrostatic and hydrophobic interactions that can also occur with mammalian cells. While these interactions are typically weaker than those between polymer and bacteria, antimicrobial efficacy and cytotoxicity need to be balanced carefully to ensure good therapeutic potential [10, 16]. This can be realized through three main strategies: First, the polymer's chemical composition can be adjusted by combining different cationic, hydrophobic and hydrophilic groups in varying ratios [18, 19, 20, 21]. Second, the polymer structure can be varied by controlling the placement of the individual functionalities, e.g., in the main chain, as same center side groups, or as different center side groups [16, 22]. Third, polymer topology can be used to control antimicrobial activity and cytotoxicity, i.e., by changing linear polymers to star polymers, hyperbranched polymers, block copolymers or cyclic polymers [19, 23].

Conventionally, optimization of chemical composition is attempted by combining hydrophobic groups with nitrogen‐based cations that mimic the respective amino acid side groups in HDPs. Examined cations include primary amines, quaternary ammonium salts (QAS), guanidinium, and imidazolium groups [24]. As alternative, tertiary sulfonium cations (TSCs) recently evolved as cations with unique characteristics. The high polarizability of sulfur and the TSCs' reduced steric hindrance (fewer bonds than QAS or quaternary phosphonium groups [25, 26, 27]) can enhance their interactions with bacterial membrane phospholipids [25]. These advantages have driven sulfonium incorporation into both main‐chain [11, 13, 17, 24, 28, 29] and side‐chain polymers [10, 13, 15, 25] for fine‐tuned antimicrobial activity.

To assess the full therapeutic potential of these antimicrobial polymers, accurate structure‐property relations are required that describe the combined influence of chemical composition and varying chain structures. Thus, we recently investigated these parameters in side‐chain sulfonium‐based random copolymers. Here, we combined sulfonium cations with different hydrophobic (aliphatic/aromatic) and hydrophilic polyethylene glycol (PEG) groups [10]. For all compositions, we systematically compared same center and different center structures and found that same center polymers are more active. Also, sulfonium‐based AMPs show superior bactericidal activity and selectivity when compared to their quaternary ammonium cationic analogues. Most importantly, we demonstrated that the additional incorporation of neutral hydrophilic PEG side chains can mitigate cytotoxicity while retaining antimicrobial activity. This concept is well‐established for ammonium‐based AMPs but requires careful and tedious optimization of terpolymer structures whenever new antimicrobial polymers are developed.

As alternative, block copolymer architectures (BCPs)[30, 31] can be used to structurally separate cationic and hydrophobic functions from the neutral hydrophilic groups. In such structures, cytotoxicity can be reduced by a discrete hydrophilic block, for example from polyethylene glycol‐based monomers [31, 32] or glycomonomers [33]. However, these blocks could also compromise antimicrobial activity [31]. Thus, their shielding influence needs to be balanced against the activity of the block which contains the cationic and hydrophobic groups. While this is established for ammonium‐based AMPs, the influence of a block copolymer structure on the sulfonium‐based side chain polymers is less explored and structure‐property relations are missing.

**FIGURE 1:**
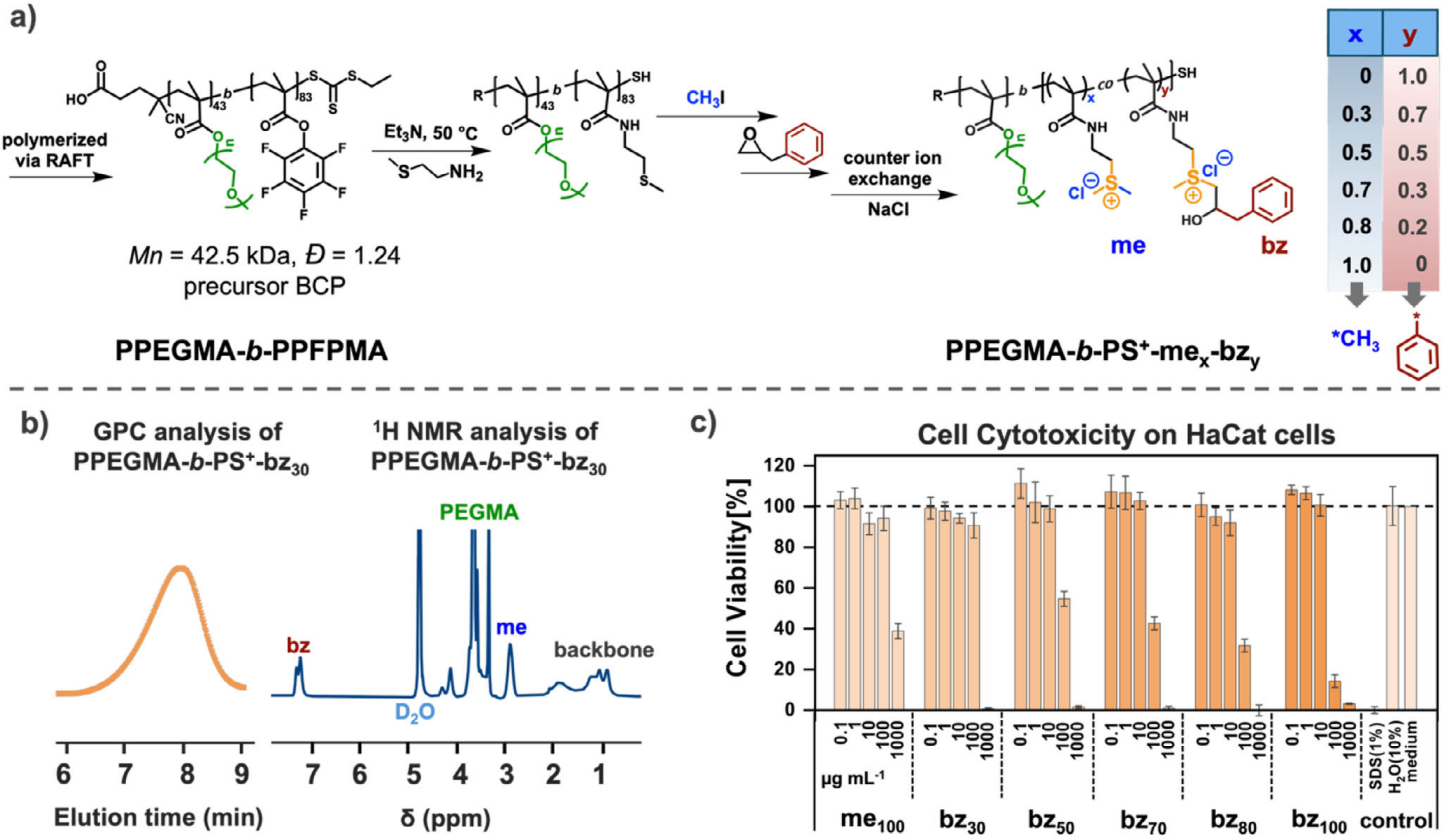
Post‐polymerization functionalization of a reactive precursor block copolymer gives a library of well‐defined sulfonium‐based BCPs with controllable hydrophobicity, narrow dispersity and good cellular compatibility. (a) Sulfonium BCPs are synthesized via a two‐step functionalization of PFP ester units in PPEGMA‐*b*‐PPFPMA, which itself is synthesized by RAFT block copolymerization. First, thioether groups are installed to generate PPEGMA‐*b*‐PMTEMAA. Second, thioethers are further functionalized with benzyl epoxides and methyl iodide to produce sulfonium cation containing polymers. Changing the ratio of *me* and *bz* hydrophobic side groups allows controlling the hydrophobicity of the BCPs to study structure‐property relations on the PPEGMA‐*b*‐PS^+^‐bz_y_ polymers. Here, me_x_ is omitted in the sample name, since the me content is determined by the corresponding bz content. (b) Exemplary GPC trace and ^1^H‐NMR spectrum of the BCP with 30 mol% bz groups show good control over the polymer structure and composition. (c) Cell viability assays on cultured human keratinocytes (HaCat) show no pronounced cytotoxic effects up to 100 µg mL^−1^ for PPEGMA‐*b*‐PS^+^‐me_100_ and PPEGMA‐*b*‐PS^+^‐bz_30_, which suggests their good cellular compatibility. Cell viability is presented as percentage with respect to the medium and as mean ± SD (n = 3).

To address this gap, we investigated the influence of a block copolymer architecture in side‐chain sulfonium‐based polymers. For this purpose, we examined the effect of two different blocks: a first block of poly(polyethylene glycol methyl ether methacrylate) (PPEGMA) as the hydrophilic block and a second block of polymethacrylamides with tunable sulfonium side groups. The PPEGMA block with a fixed molecular weight was incorporated to maintain a consistent hydrophilic shielding effect across all polymers. To control the AMP's hydrophobicity, the carbon‐to‐cation ratio was varied in the active sulfonium‐based block. This was achieved by systematically functionalizing the sulfonium cations with benzyl (*bz*) and methyl (*me*) groups. Varying the *bz*:*me* ratio in the active block resulted in BCPs with controllable composition but a consistent overall chain structure, i.e., same chain length and dispersity for both blocks. With this approach, we prepared a library of six distinct BCPs (see Figure 1a) to establish structure‐property relations that could guide the development of more effective and selective antimicrobial block copolymers.

## 2 Results and Discussion

To create the BCP library, we used a two‐step post‐polymerization functionalization of a reactive precursor block copolymer, i.e., poly(polyethylene glycol methyl ether methacrylate‐*b*‐pentafluorophenyl methacrylate) (PPEGMA*‐b*‐PPFPMA) (see Figure 1a). This precursor master batch was the foundation for all final sulfonium BCPs, thereby minimizing batch‐to‐batch inconsistencies in degree of polymerization (DP) and dispersity (*Ð*).

To control the polymer structure, we used reversible addition‐fragmentation transfer (RAFT) polymerization. This method allowed us to prepare a well‐defined precursor BCP with blocks of similar molecular weights. For the first block, PEGMA_500_ was polymerized using 4‐cyano‐4‐[(ethylsulfanylthiocarbonyl)sulfanyl] pentanoic acid, as chain transfer agent (CTA) (see ESI for experimental details). The resulting PPEGMA homopolymer then served as a macro‐CTA for chain extension with pentafluorophenyl methacrylate (PFPMA) to form the precursor block copolymer PPEGMA‐*b*‐PPFPMA (see ESI for experimental details). The successful synthesis of the precursor BCP was confirmed through gel permeation chromatography (GPC) and nuclear magnetic resonance (NMR) spectroscopy. GPC analysis of the PPEGMA block showed a number‐average molecular weight (*M_n_*) of 18 000 g mol^−1^ with low dispersity (*Đ* = 1.22), suggesting controlled polymerization. After chain extension, the BCP exhibited a higher molecular weight (*M_n_* = 31 000 g mol^−1^) with narrow monomodal distribution (*Đ* = 1.24) (ESI, Figure S1), demonstrating successful synthesis of a well‐defined BCP. For precise molecular weight determination via monomer conversion, we used ^1^H‐NMR spectroscopy with DMF as internal standard (ESI, Figure S2). The PPEGMA block showed 83% conversion, corresponding to 43 repeating units and a corresponding molecular weight of 21 500 g mol^−1^. The PPFPMA block exhibited 72% conversion, corresponding to 83 repeating units and a molecular weight of 21 000 g mol^−1^ (ESI, Figure S3). This yielded an overall molecular weight of 42 500 g mol^−1^. Characteristic NMR peaks for both blocks further confirmed the PPEGMA_43_‐*b*‐PPFPMA_83_ structure (ESI, Figure S3), with each block contributing approximately 21 kDa to the final polymer architecture.

The final sulfonium‐based block copolymers were synthesized through a two‐step functionalization of PPEGMA‐*b*‐PPFPMA (Figure 1a). In the first step, substitution of PFP esters with 2‐(methylthio)ethylamine introduced pendant thioether groups, i.e., transforming the PPFPMA block into poly(*N*‐(2‐(methylthio)ethyl)methacrylamide) (PMTEMAA) and generating PPEGMA‐*b*‐PMTEMAA. PFP‐ester conversion was verified by disappearance of all fluorine signals in ^19^F‐NMR (ESI, Figure S4). GPC analysis confirmed the block copolymer maintained the precursor's well‐defined architecture. The preserved molecular weight distribution further hints towards limited disulfide formation of the thiol polymer end groups that result from aminolysis during the first functionalization. (ESI, Figure S5, comparing PPEGMA‐*b*‐PMTEMAA and PPEGMA‐*b*‐PPFPMA). Finally, ^1^H‐NMR spectra revealed new peaks at 2.78 – 2.44 ppm, with end group analysis suggesting complete thioether functionalization (ESI, Figure S6). In the second step, thioether units in the PMTEMAA block were functionalized with methyl iodide and benzyl epoxide to generate tertiary sulfonium cations (see ESI for synthetic details, Figure S7). In this reaction, a first addition of methyl iodide partially methylates the thioether units to give a targeted fraction of dimethyl‐based sulfonium cations. Subsequent addition of benzyl epoxide functionalizes the remaining thioether units to give tertiary sulfonium cations with methyl and benzyl side groups on the same sulfur center. To ensure comparability with established chloride‐containing AMPs, the TFA and iodide counterions were exchanged to chlorides via dialysis of the crude BCPs against aqueous NaCl. (ESI, Figure S8).

To systematically investigate the effect of BCP hydrophobicity, we functionalized the PMTEMAA block with varying ratios of methyl iodide and benzyl epoxide. These two functionalities (*me* and *bz* side groups) were selected due to their distinct differences in bioactivity [10]: In our previous study, we found that sulfonium polymers with *me* side groups (PS^+^‐me) show optimal selectivity but limited antimicrobial activity. On the other hand, *bz* side group containing polymers (PS^+^‐bz) exhibit excellent bactericidal activity but toxic side effects. Based on these extremes, we assume that combining methyl and benzyl groups in different molar proportions can be used to balance their opposing bioactivities in the active block of our BCP.

Testing this hypothesis, we created a BCP library with varying hydrophobic group composition but consistent cation density and polymer architecture. This library is based on varying benzyl contents from 0 mol% to 100 mol%, i.e., from purely methyl‐functionalized PPEGMA‐*b*‐PS^+^‐me_100_ (me_100_) to PPEGMA‐*b*‐PS^+^‐bz_100_ (bz_100_) polymers, where every sulfonium cation contains one benzyl group. Additional *bz* contents are 30, 50, 70, and 80 mol% and polymers are denoted as PPEGMA‐*b*‐PS^+^‐bz_30_ (bz_30_), PPEGMA‐*b*‐PS^+^‐bz_50_ (bz_50_), PPEGMA‐*b*‐PS^+^‐bz_70_ (bz_70_), PPEGMA‐*b*‐PS^+^‐bz_80_ (bz_80_), respectively (Figure 1b and ESI, Table S1). In the sample names, *me_x_* is omitted since the *me* content is determined by the corresponding *bz* content. The successful synthesis of all sulfonium‐based BCPs was demonstrated by ^1^H‐NMR spectroscopy. Quantitative peak analysis revealed complete conversion of thioethers to sulfoniums as well as the relative degrees of functionalization with *me* and *bz* groups (ESI, Figures S9–S14, Table S3). Additionally, GPC analysis showed a systematic increase in molecular weight with higher *bz* content compared to *me*‐only functionalized polymers. This is suggested to result from bulkier *bz* groups (ESI, Figure S15). However, the dispersity of all final sulfonium BCPs remained comparable to the precursor BCP, thus suggesting successful retention of well‐defined architectures throughout the functionalization process.

The potential of these polymers for therapeutic antimicrobial applications depends on their cytotoxicity against human cells. For this, viability of HaCat and L929 cells were determined at polymer concentrations of 0.1, 1, 10, 100, and 1000 µg mL^−1^ (see ESI for further details). For the most hydrophilic polymers, PPEGMA‐*b*‐PS^+^‐me_100_ and PPEGMA‐*b*‐PS^+^‐bz_30_, no cytotoxic effects were observed for polymer concentrations up to 100 µg mL^−1^ (see Figure 1c). However, for polymers with *bz* contents ≥ 50 mol%, cell viability at 100 µg mL^−1^ decreased drastically (see Figure 1c; Figure S16). These findings indicate a correlation between hydrophobicity and cytotoxicity that needs to be considered when assessing their therapeutic potential.

**FIGURE 2:**
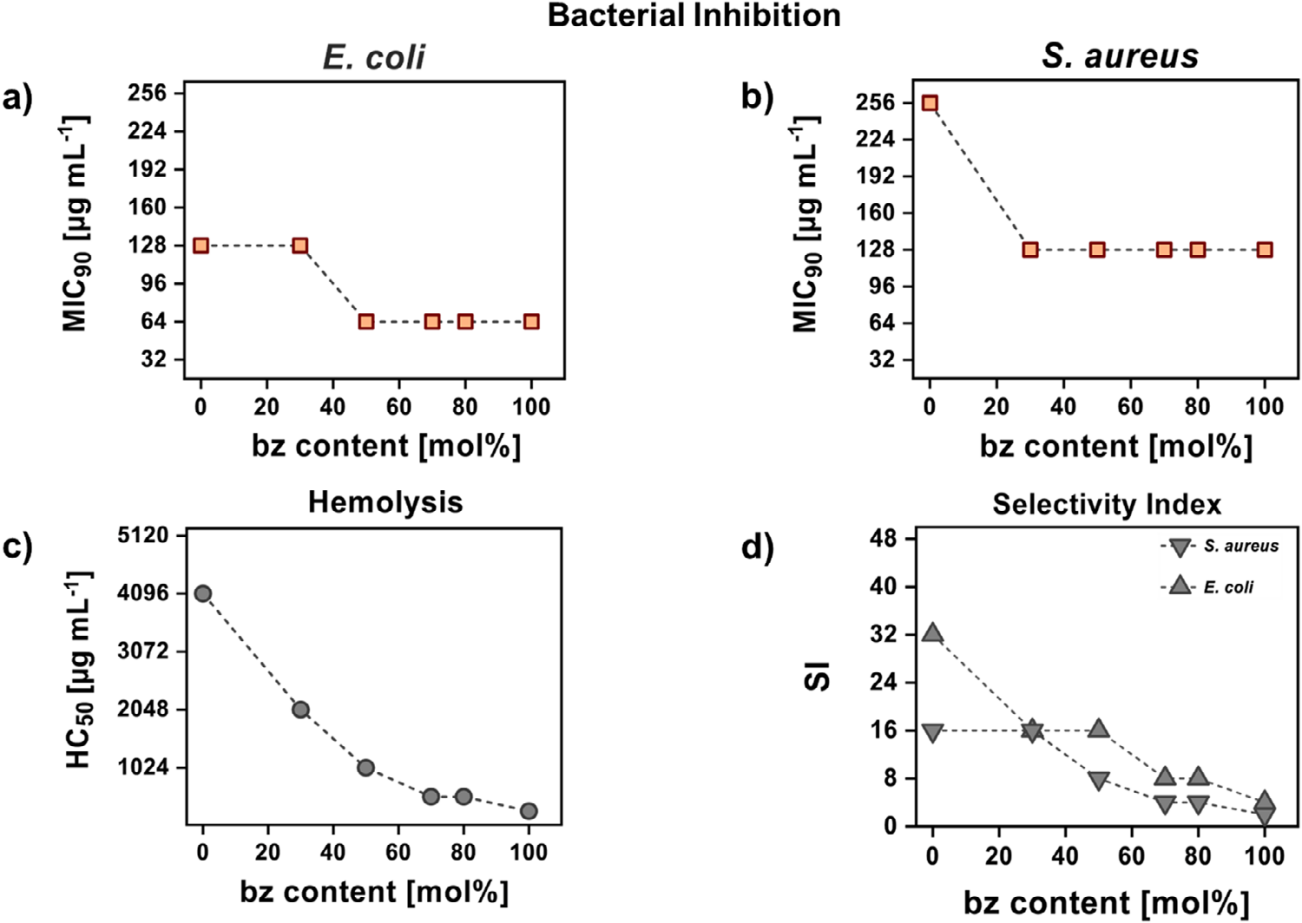
Bacterial growth inhibition and hemolytic activity of PPEGMA‐*b*‐PS^+^‐bz_y_ sulfonium BCPs increase with their hydrophobicity, i.e., their *bz* content in percent (bz_y_). (a, b) Antimicrobial activity against *E. coli* and *S. aureus* increases with increasing benzyl content of sulfonium BCPs. This is represented by a decreasing MIC_90_. (c) Hemolytic activity increases with *bz* content, which causes a decrease in HC_50_. (d) The selectivity towards bacterial cells decreases with increasing *bz* content. This is demonstrated by a decrease in selectivity index (SI), which is determined as ratio between HC_50_ and MIC_90_.

Next, we determined two key properties: (i) their antimicrobial activity and (ii) their hemolytic activity. The balance between these properties is critical, as it determines the polymers' efficiency in inhibiting bacterial growth while minimizing toxic side effects. To systematically investigate the influence of polymer structure and composition on these parameters, the following sets of experiments were conducted:

First, the antimicrobial activity of all polymers was tested against Gram‐positive and Gram‐negative bacteria to determine the susceptibility of different bacterial cell walls. *E. coli* and *S. aureus* were selected as representative strains for Gram‐negative and Gram‐positive bacteria, respectively. To quantitatively assess the antimicrobial activity of the polymers, a standard broth micro‐dilution method was conducted. This gave access to the minimum inhibitory concentration, which is defined as the lowest polymer concentration inhibiting more than 90% bacterial growth (MIC_90_) [10, 15]. All the BCPs were active against the tested strains and MIC_90_ decreased with polymer hydrophobicity, i.e., as *bz* content increases (Figure 2a,b). Against *E. coli*, the polymers with low benzyl contents of 0 mol% (PPEGMA‐*b*‐PS^+^‐me_100_) and 30 mol% (PPEGMA‐*b*‐PS^+^‐bz_30_) showed the highest MIC_90_ of 128 µg mL^−1^. The polymers with *bz* contents ≥ 50 mol% showed an increased activity with MIC_90_ values of 64 µg mL^−1^ (Figure 2a). For *S. aureus*, the most hydrophilic polymers me_100_ showed a lower activity with a MIC_90_ around 256 µg mL^−1^. More hydrophobic polymers with *bz* contents ≥ 50 mol% were more active with MIC_90_ of 128 µg mL^−1^ (Figure 2b). Overall, the slightly reduced inhibitory effect against *S. aureus* is assigned to the outer peptidoglycan layer in Gram‐positive bacteria, which is less susceptible to the interactions with AMPs than the lipopolysaccharide outer layer of Gram‐negative bacteria [34].

Second, hemolytic activity of the polymers was tested against isolated fresh sheep erythrocytes. A standard hemoglobin release assay was used to assess the influence of polymer concentration on hemolysis and determine the hemolytic concentration at which 50% hemolysis occurred (HC_50_) (see ESI for experimental details). For the most hydrophilic polymers, PPEGMA‐*b*‐PS^+^‐me_100_ and PPEGMA‐*b*‐PS^+^‐bz_30_, hemolytic activity is low with HC_50_ above 4096 and 2048 µg mL^−1^, respectively (Figure 2c). However, increasing polymer hydrophobicity increased the hemolytic activity, i.e., HC_50_ decreased with an increasing *bz* content. For *bz* contents ≥ 50 mol%, interaction of *bz* groups with mammalian cell membranes causes significant hemolytic effects and HC_50_ is reduced to around 512 – 256 µg mL^−1^. Thus, *bz* contents ≤ 30 mol% are required to prevent toxic side effects in sulfonium‐based amphiphilic block polymers.

Estimating an overall therapeutic potential requires to weigh both effects against each other. For this, the selectivity index (SI) is used as the ratio of HC_50_ to MIC_90_ [35]. As shown in Figure 2d, selectivity is higher for hydrophilic polymers with low *bz* contents: The SI values for PPEGMA‐*b*‐PS^+^‐me_100_ and PPEGMA‐*b*‐PS^+^‐bz_30_ were higher than those for BCPs with *bz* contents ≥ 50 mol%. These findings support the results from the cytotoxicity tests that revealed a decrease in cell viability for BCPs with *bz* contents ≥ 50 mol% at 100 mg mL^−1^ (see Figure 1c). Consequently, this data suggests a threshold hydrophobicity (30 – 40 mol% *bz* content) above which the membrane disrupting potential does not differentiate between bacterial and mammalian cells. Thus, BCPs below this threshold would be considered promising therapeutics since toxic effects at the required therapeutic concentration (MIC_90_) are justifiable. However, absolute values for selectivity and antimicrobial activity below this threshold are relatively modest: PPEGMA‐*b*‐PS^+^‐bz_30_ shows SI values of 16 and MIC_90_ of 128 mg mL^−1^ against *E. coli* and *S. aureus*. Comparing these values to those of their random copolymer analogues suggests that the BCP architectures are intrinsically less active but show comparable cytotoxicity [10].

However, these moderate membrane‐disrupting capabilities present a versatile opportunity to enhance the bacterial uptake of conventional small molecule antibiotics. In such a therapeutic strategy, the polymers do not act alone but rather serve as adjuvants that permeabilize bacterial membranes [36]. As a result, a lower antibiotic dose is required, synergistic antimicrobial effects can be created, and resistance mechanisms can be mitigated. This approach also has proven effective against multidrug‐resistant bacteria [37] and is particularly promising to enhance the efficacy of antibiotics with prominent side effects that would otherwise vanish from the clinics, e.g., ciprofloxacin [36, 37].

To evaluate the ability of our BCPs to enhance the efficacy of conventional antibiotics, we combined them with two commercially available antibiotics, penicillin G (Pen G) and ciprofloxacin (Cipro) (Figure S17). These antibiotics were selected based on their different bacterial selectivity and mechanisms of action: Cipro inhibits bacterial DNA synthesis and is more effective against Gram‐negative bacteria, while Pen G disrupts cell wall biosynthesis and is more effective against Gram‐positive bacteria. Thus, we evaluated whether a combination with sulfonium‐BCPs can enhance their efficacy against the type of bacteria that is conventionally less susceptible to the antibiotic alone. Similarly, we selected two polymers that show different activities against the two bacterial strains: PPEGMA‐*b*‐PS^+^‐bz_80_ (bz_80_), which shows better activity against Gram‐negative *E. coli* and PPEGMA‐*b*‐PS^+^‐bz_30_ (bz_30_), which shows similar activities against *E. coli* and Gram‐postive *S. aureus*.

Based on these considerations, we designed a combination matrix to systematically examine the influence of polymer structure and antibiotic on the combined activity against different bacteria: Each polymer (bz_80_ and bz_30_) was combined with each antibiotic (Pen G and Cipro) and tested against each bacterial strain (*E. coli* and *S. aureus*). With this, we paired polymers, that perform better against one strain, with antibiotics, that perform low against this strain and vice versa. As controls, we included combinations of polymers and antibiotics that show similar preferences against bacterial strains. This design allowed us to systematically probe potential synergies while accounting for the differential activities.

**FIGURE 3:**
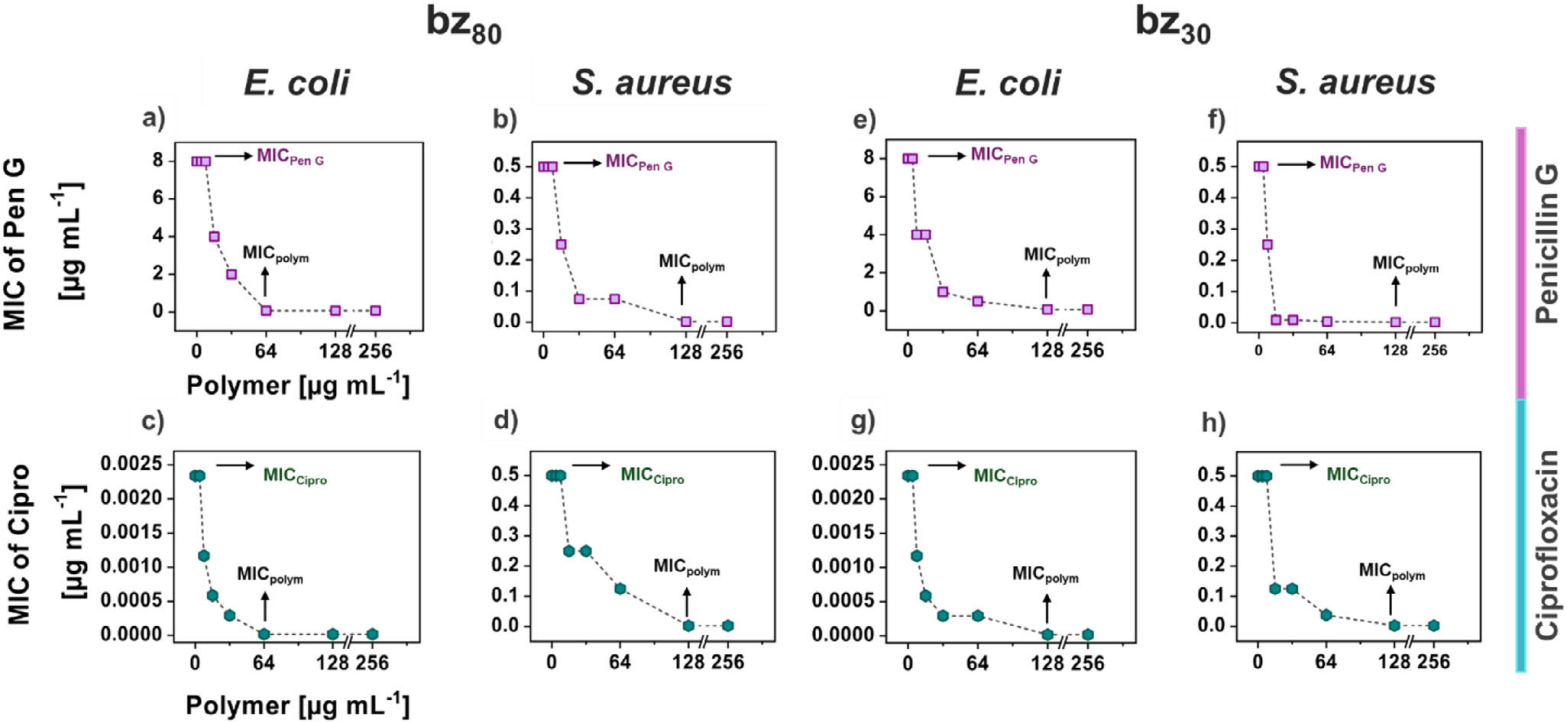
Sulfonium‐based BCPs (bz_30_ and bz_80_) enhance antibiotic activity of penicillin G (Pen G) and ciprofloxacin (Cipro) below the MIC_90_ of the polymers. This is demonstrated by the influence of bz_80_ and bz_30_ concentration on the antibiotics’ MIC_90_ against *E. coli* and *S. aureus*: For bz_80_, MIC_90_ of Pen G, decreases with increasing polymer concentrations against (a) *E. coli* and (b) *S. aureus*. MIC_90_ of Cipro decreases with increasing concentrations of bz_80_ against (c) *E. coli* and (d) *S. aureus*. For bz_30_, the MIC_90_ of Pen G decreases with increasing polymer concentration against (e) *E. coli* and (f) *S. aureus*. Increasing bz_30_ concentration also increases antibiotic activity of Cipro against (g) *E. coli* and (h) *S. aureus*.

In a first set of experiments, we focused on PPEGMA‐*b*‐PS^+^‐bz_80_ (bz_80_) since its higher membrane disrupting potential is assumed to facilitate synergistic effects with antibiotics. In a bacterial growth assay, we determined the MIC_90_ of the antibiotics in the presence of different polymer concentration (see ESI for experimental details). We found that bz_80_ was able to reduce the MIC_90_ of Pen G and Cipro against both bacterial strains. Importantly, this reduction was observed for polymer concentrations below the respective MIC_90_ of the polymer alone. In combination with Pen G, bz_80_ reduced the MIC_90_ against *E. coli* by 4‐fold, from 8 µg mL^−1^ to 2 µg mL^−1^ (Figure 3a). Against *S. aureus*, bz_80_ reduced the MIC_90_ of Pen G by 8‐fold, from 0.5 µg mL^−1^ to 75 ng mL^−1^ (Figure 3b). When combined with Cipro, bz_80_ reduced the MIC_90_ against *E. coli* by 8‐fold from 2.34 to 0.2875 ng mL^−1^ (Figure 3c) and against *S. aureus* by 4‐fold from 0.5 to 0.125 µg mL^−1^ (Figure 3d).

Based on these results, we examined whether similar effects could be achieved by a BCP with better hemocompatibility. For this, we tested PPEGMA‐*b*‐PS^+^‐bz_30_ (bz_30_), which exhibits balanced antibacterial activity and low hemolytic toxicity (SI = 16 against *E. coli* and *S. aureus*). Moreover, its excellent cell viability at concentrations up to 100 µg mL^−1^ further supports its suitability for biological applications. Combining bz_30_ with Pen G and Cipro revealed that the BCP significantly reduces the MIC_90_ of these antibiotics. Again, this was observed for polymer concentrations below the MIC_90_ of bz_30_. In combination with Pen G, bz_30_ reduced the MIC_90_ of the antibiotic against *E. coli* by 16‐fold from 8 to 0.5 µg mL^−1^ (Figure 3e). At the same sub‐MIC level, bz_30_ reduced the MIC_90_ of Pen G against *S. aureus* by 100‐fold from 0.5 µg mL^−1^ to 4.86 ng mL^−1^ (Figure 3f). Similar effects were observed for combinations of bz_30_ and cipro, though to a lesser extent than with Pen G. For *E. coli*, bz_30_ reduced the MIC_90_ of Cipro by 8‐fold from 2.34 to 0.2875 ng mL^−1^ (Figure 3g). Against *S. aureus*, a 16‐fold reduction in the MIC of cipro was observed at the same sub‐MIC level, from 0.5 µg mL^−1^ to 37.5 ng mL^−1^ (Figure 3h). Lower sub‐MIC concentrations of bz_30_ showed reduced effects. Overall, these results demonstrate that both BCPs (bz_80_ and bz_30_) can effectively lower the required dose of conventional antibiotics.

**FIGURE 4:**
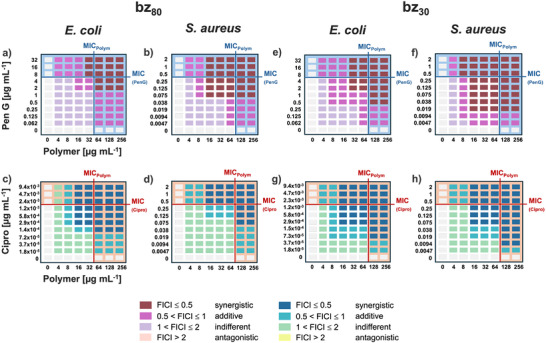
Combinations between sulfonium‐based BCPs and penicillin G (Pen G) or ciprofloxacin (Cipro) show synergistic effects against *E. coli* and *S. aureus*. Combination effects are determined by checkerboard microdilution analysis and calculated as fractional inhibitory concentration index (FICI). FICI values are represented as linear color gradient in dependence of polymer‐ and drug concentration. Synergistic effects below MIC_90_ of antibiotic alone and polymer alone occur for two scenarios: First, bz_30_ and bz_80_ both enhance the efficacy of antibiotics against their preferred strains, i.e., against bacterial types that are particularly susceptible to this antibiotic. Synergistic interactions occur with Pen G against Gram‐positive *S. aureus* (b, f) and with Cipro against Gram‐negative *E. coli* (c, g). Second, polymers increase the efficacy of antibiotics against bacteria that are less susceptible to this drug, e.g., Pen G against *E. coli* and Cipro against *S. aureus*. A pronounced difference between bz_30_ and bz_80_ is observed and synergistic effects are found for bz_30_ also with Pen G towards *E. coli* (e) and with Cipro against *S. aureus* (h). In contrast, no comparable effects against the non‐preferred strains were observed for bz_80_., i.e., no synergy with Pen G against *E. coli* (a) and not with Cipro against *S. aureus* (d).

Interestingly, pronounced differences were observed between the combinations of strains, polymers, and antibiotics. To distinguish between actual synergistic or mere additive effects between BCP and antibiotic, we evaluated the MIC data via checkerboard analysis [37]. For these plots, fractional inhibitory concentration indexes (FICIs) were calculated for every possible combination of polymer and drug concentration (see ESI for details). FICI is a parameter generally used to determine the synergistic/antagonistic and additive interactions of compounds in combination therapy. The FICI values were interpreted as follows: FICI ≤ 0.5 indicated a synergistic effect, 0.5 < FICI ≤ 1 suggested additive, 1 < FICI ≤ 2 indicated indifference, and >2 represented an antagonistic effect. The resulting FICI values are represented in a heat map as function of polymer and antibiotic concentration in Figure 4.

Overall, two different scenarios can be distinguished: First, polymers can enhance the efficacy of antibiotics against their preferred strains, i.e., against bacterial types that are particularly susceptible to this antibiotic. Here, both polymers showed distinct synergistic interactions with Pen G against Gram‐positive *S. aureus* (Figure 4b, f) and with Cipro against Gram‐negative *E. coli* (Figure 4c, g). Differences in the effects of bz_30_ and bz_80_ are small with slightly more synergistic combinations observed for bz_30_. Particularly, bz_30_ showed strong synergistic interactions for 15 different combinations with Pen G against *S. aureus* (Figure 4f). In comparison, the bz_80_ polymer exhibited more indifferent interactions (1 < FICI ≤ 2) with both antibiotics against the tested bacterial strains. However, none of the combinations in both polymers demonstrated antagonistic effects (FICI > 2).

Second, polymers can also increase the efficacy of antibiotics against bacterial strains that are conventionally less susceptible to this antibiotic. For example, Pen G against *E. coli* and Cipro against *S. aureus*. In this case, a pronounced difference between bz_30_ and bz_80_ is observed and synergistic effects are only found for the bz_30_ polymer. For instance, Pen G is more active towards *S. aureus* but when combined with bz_30,_ synergy was found against *E. coli* for 4 different combinations (Figure 4e). Similarly, Cipro is more effective against *E. coli* but showed significant synergy against *S. aureus* when tested in combination with bz_30_ (Figure 4h). In contrast, no comparable synergistic effects against the non‐preferred strains were observed for bz_80_., i.e., bz_80_ did not show synergistic effects with Pen G against *E. coli* (Figure 4a) and not with Cipro against *S. aureus* (Figure 4d).

In an overall comparison of both polymers, it is noteworthy that bz_30_ showed synergistic antibacterial activity regardless of the strain, i.e., this polymer was able to induce synergy even if the antibiotic was conventionally less susceptible to the tested strain. In contrast, bz_80_ only induced synergistic effects with antibiotics that already show high activity toward a specific strain by themselves. We assume that this reduced performance of the bz_80_ polymer is the result of different physical properties of the polymer‐antibiotic combinations in aqueous solutions. Particularly, we assume that the more hydrophobic bz_80_ polymer can form BCP micelles or polyplexes with the antibiotics. Such self‐assembled structures would constrain the active sulfonium blocks in the micellar core, thereby reducing their interaction potential with bacterial membranes [30].

To test this hypothesis, we examined potential micellization of all BCPs via GPC. For this, polymers were dissolved at a concentration of 3 mg mL^−1^ in Milli‐Q water and subjected to GPC analysis. The elugrams for bz_80_ and bz_100_ showed a shoulder at lower elution times, thus suggesting the presence of higher molecular weight assemblies, e.g. micelles (ESI, Figure S18). These micelles were not observed in GPC measurements at lower polymer concentrations of 1.0 mg mL^−1^, confirming concentration‐dependent micellization of the isolated BCPs (ESI, Figure S15). While this self‐assembly requires high polymer concentrations, we assume that potential electrostatic interactions between the polymers’ cationic sulfonium blocks and the antibiotics’ carboxylate functionalities can lead to the formation of polyplexes at lower polymer concentrations. To test this assumption, we investigated potential polyplex formation in the presence of Pen G through dynamic light scattering (DLS). For this purpose, all different polymers were mixed with Pen G, as exemplary antibiotic, in a 1: 1 molar ratio in Milli‐Q water at different total concentrations. Then, scattering intensity of resulting samples was measured via DLS and plotted against the polymer concentration (ESI, Figure S19). For all polymers, a two‐step profile was observed where a rapid increase in scattering intensity at a specific polymer concentration suggests the formation of micelles. From these data, we determined the critical micelle concentration (CMC) for each polymer (ESI, Figure S20) and found that CMC decreases linearly with bz content. This suggests a combined influence of electrostatic and hydrophobic interactions on polyplex formation. Comparing CMC values for bz_30_ and bz_80_, it can be seen that the more hydrophobic bz_80_ forms polyplexes with Pen G at lower concentrations (CMC = 0.55 mg mL^−1^) than the more hydrophilic bz_30_ (CMC = 1 mg mL^−1^). These findings support our hypothesis that interactions between polymer and antibiotic might hinder synergistic effects for the more hydrophobic bz_80_ polymer. We suggest that such polymer‐antibiotic interactions might still be present at concentrations below the CMC that are used in the antibacterial assays. Overall, these results demonstrate the importance of accurate structure‐property relationships in designing new antimicrobial materials.

## 3 Conclusion

In conclusion, this study shows the potential of sulfonium‐based amphiphilic block copolymers (BCPs) as adjuvants for antimicrobial therapy. By combining a hydrophilic PPEGMA block with a cationic sulfonium‐containing block, we demonstrate how precise structural modulation, through variation in hydrophobic side groups (*me* and *bz*), can balance antimicrobial efficacy and cytocompatibility in BCP architectures. These findings complement our previous studies on random copolymers and show that the diblock architecture significantly reduces hemolytic toxicity but also antibacterial activity, particularly in compositions with lower hydrophobic contents. However, despite the lower absolute antimicrobial activity, such BCPs retained membrane‐disruptive potential, which enabled their use as adjuvants in combination with conventional small‐ molecule antibiotics such as penicillin G and ciprofloxacin. Synergistic effects were particularly pronounced for PPEGMA‐*b*‐PS⁺‐bz_30_, which showed broad‐spectrum activity. In contrast, DLS studies suggest that increased polyplex stability between bz_80_ and Pen G could reduce antibiotic availability for the more hydrophobic BCP, thus linking polymer self‐assembly behavior to therapeutic performance.

These results highlight the impact of polymer architecture on controlling synergistic effects with antibiotics and the developed structure‐property relations provide a framework for the rational design of antimicrobial polymer–drug combinations. Therefore, this work contributes to an emerging strategy of using functional polymers to restore or enhance antibiotic potency.

Looking forward, future research should explore the in vivo efficacy and safety of these polymers, expand the chemical diversity of the block components, and evaluate their performance against multi‐drug‐resistant pathogens. Such efforts will be critical for translating these promising materials into therapeutic agents capable of addressing the escalating global threat of AMR.

## 4 Experimental Part

### 4.1 Materials and Methods

Please see ESI for detailed information on materials and methods used for this research work.

### 4.2 Synthesis of Antimicrobial Polymers

#### 4.2.1 Synthesis of Precursor Polymers

##### 4.2.1.1 Synthesis of poly(polyethylene glycol methacrylate) (PPEGMA)

The PPEGMA homopolymer was synthesized as macro‐CTA. For this, we used a modified literature procedure [38]. In brief, a mixture of PEGMA (10 g, 20 mmol, 52 eq.), 4‐cyano‐4‐[(ethylsulfanylthiocarbonyl) sulfanyl]pentanoic acid (CTA) (106 mg, 383 µmol, 1.0 eq.), and 4,4'‐Azobis(4‐cyanovaleric acid) (ACVA) (12 mg, 42 µmol, 0.125 eq.) was dissolved in 20 mL of anhydrous dioxane. Anhydrous dimethylformamide (DMF) (1 mL) was added as an internal standard. The reaction mixture was purged with nitrogen for 30 min, after which the flask was sealed and placed in a pre‐heated oil bath at 65°C for 16 h. Please see ESI for information on its nuclear magnetic resonance (NMR) and gel permeation chromatography (GPC) analysis for monomer conversion.

##### 4.2.1.2 Synthesis of PPEGMA‐b‐PPFPMA

The PPEGMA‐*b*‐PPFPMA block copolymer was synthesized using a modified literature procedure [38]. A mixture of PPEGMA macro‐CTA (5 g, 227 µmol, 1.0 eq.), PFPMA (6.86 g, 27.24 mmol, 120 eq.), and ACVA (7.96 mg, 28.4 µmol, 0.125 eq.) was dissolved in 25 mL of anhydrous dioxane, with 1 mL of anhydrous DMF added as an internal standard. The reaction vessel was purged with nitrogen for 30 minutes, sealed, and heated in an oil bath at 80°C for 16 hours. Please see ESI for information on its NMR and GPC analysis for monomer conversion.

#### 4.2.2 Post‐Polymerization Functionalization of Precursor Polymer PPEGMA‐b‐PPFPMA

##### 4.2.2.1 1^st^ Functionalization of PFPMA Units to Thioether Side Group

PPEGMA‐*b*‐PPFPMA was functionalized using our previously established protocol [10]. Please see ESI for specific details on synthesis and quantitative functionalization via NMR.

##### 4.2.2.2 2^nd^ Functionalization to sulfonium polymers

The thioether polymer acts as a platform for secondary functionalization. In this step, benzyl epoxide and methyl iodide were employed to simultaneously introduce sulfonium cations as side groups and control hydrophobicity by varying the ratio of methyl (*me*) and benzyl (*bz*) groups.

For the synthesis of PPEGMA‐*b*‐PS^+^‐bz_100_, a previously established protocol was followed [10]. For the remaining polymers in the library, a modified protocol was used. In cases where both benzyl and methyl functional groups were incorporated, (2,3‐epoxypropyl)benzene and methyl iodide were added in varying ratios (as detailed in Table S1, ESI).

In a representative reaction for synthesizing PPEGMA‐*b*‐PS^+^‐bz_y_, the thioether polymer was dissolved in DMF and heated to 50°C. Methyl iodide (CH_3_I) was added, and the mixture was stirred overnight. Subsequently, (2,3‐epoxypropyl)benzene and trifluoroacetic acid (TFA) were added, and the reaction was stirred for an additional day. For PPEGMA‐*b*‐PS^+^‐me_100_, the reaction was conducted in DMF at 50 °C using only an excess of CH_3_I (12 equivalents).

After the reaction was ended, the functionalized polymer was purified through dialysis (please see ESI for information on purification and NMR analysis of BCPs).

### 4.3 Biological Tests

#### 4.3.1 Cytotoxicity Assay

Cytotoxicity of the BCPs was evaluated on L929 and HaCat cells using a previously established protocol [10] (please see ESI for more details).

#### 4.3.2 MIC Determination via Broth Dilution Assays

A previously established procedure was followed to determine antimicrobial activity of individual BCPs [10, 13, 15] (please see ESI for detailed information). However, synergistic MIC assay was performed by selecting bz_30_ and bz_80_ polymers as representatives of a hydrophilic BCP and a more hydrophobic BCP. Their effect on activity of penicillin G and ciprofloxacin was determined against *S. aureus* and *E. coli* by using the following method: For each strain, a preculture was prepared by inoculating a glycerol stock solution in 5 mL of LB‐Medium, which was incubated overnight at 37°C with shaking at 140 rpm. The OD_600_ of the bacterial culture was measured using absorbance reader and diluted to an OD value of 0.02 before adding to the wells for experiments. For each polymer, 4 mL stock solutions with 8, 16, 32, 64, 128, 256, and 512 µg mL^−1^ were prepared in sterile Milli‐Q water in 5 mL sterile vials. 100 µL of each stock solution were added to a new empty well in a 96‐well plate. 500 µL of each polymer stock were used to prepare combined polymer‐antibiotic stock solutions: For *E. coli*, 128 µg mL^−1^ and 75 ng mL^−1^ stock solutions of penicillin G and ciprofloxacin were prepared for each polymer concentration, respectively. For *S. aureus*, 16 µg mL^−1^ stock solutions of both penicillin G and ciprofloxacin were prepared for each polymer concentration. The stock solutions of polymer‐antibiotic combinations were then used to prepare a series of different concentrations by 2‐fold dilutions (each 100 µL in 96‐well plates). For the MIC test, 100 µL of the diluted bacterial suspension (OD ≈ 0.02) were prepared and added to each well. This gave final concentrations for each antibiotic and polymer according to the dilution of their stock solutions. For instance, for Pen G, each 128 µg mL^−1^ stock solution with a different polymer concentration was diluted to further 11 concentrations i.e., 32, 16, 8, 4, 2, 1, 0.5, 0.25, 0.125, 0.075, 0.0375 µg mL^−1^. This gave combinations of each antibiotic concentration with a respective polymer concentration. For each 75 ng mL^−1^ stock solution of cipro, 18.75, 9.4, 4.68, 2.34, 1.17, 0.586, 0.293, 0.147 ng mL^−1^ concentrations were tested. For each 16 µg mL^−1^ stock solution of both Pen G and cipro, 4, 2, 1, 0.5, 0.25, 0.125, 0.075, 0.0375, 0.0188, 0.0094, 0.0047 µg mL^−1^ concentrations were tested. Positive and negative controls, without antimicrobial agent and bacteria, respectively, were also included. The plates were incubated at 37 °C for 20 h, and the absorbance at 600 nm was subsequently recorded. From here, MICs of antibiotics were calculated in combination with polymer, where polymer and antibiotics combined inhibited more than 90% of bacterial growth. The MICs of individual polymer and antibiotics were used as calculated from above MIC test. Fractional inhibitory concentration index (FICI) values were used to determine the interactions between polymer and drugs (please see ESI for information on FICI calculation).

#### 4.3.3 Hemolysis Assay

Hemolytic activity of the BCPs was evaluated using a previously established protocol [10, 39]. (please see ESI for more details).

### 4.4 Micelle Formation and CMC Determination

For micelle formation, polymer solutions in filtered Milli‐Q water were prepared at concentrations ranging from 1.25 µg mL^−1^ to 1.5 mg mL^−1^ (1.25, 2.5, 5, 10, 25, 50, 100, 250, 500, 1000, and 1500 µg mL^−1^). The solutions were stirred to ensure homogeneity and left overnight with sealed lids to prevent evaporation. The next day, Pen G was added to each vial at a 1: 1 molar ratio relative to the polymer. After incubation for 48 hrs, critical micelle concentration (CMC) of samples was determined via dynamic light scattering (DLS). Please see ESI for detailed procedure on micelle formation and CMC determination.

## Conflicts of Interest

The authors declare no conflicts of interest.

## Supporting information




**Supporting File**: marc70076‐sup‐0001‐SuppMat.docx.

## Data Availability

The data that support the findings of this study are available from the corresponding author upon reasonable request.
