# Supplementary material for: Sulfonium‐Based Antimicrobial Block Copolymers: Influence of Hydrophobicity on Biological Activity and Antibiotic Synergy
Source: Macromol Rapid Commun. 2025 Sep 30;47(14):e00421. doi: 10.1002/marc.202500421 (PMC13384801; doi:10.1002/marc.202500421)
Supplement: Supplementary file 1 — Supporting File: marc70076‐sup‐0001‐SuppMat.docx. [file MARC-47-e00421-s001.docx]

**Electronic Supplementary Information**

**Sulfonium-Based Antimicrobial Block Copolymers: Influence of Hydrophobicity on Biological Activity and Antibiotic Synergy**

*Sidra Kanwal^1^, Otto Staudhammer^1^, Umer Bin Abdul Aziz^1^, Elisa Quaas^2^, Jörg Rademann^1^, Daniel Klinger^1*^*

^1^Freie Universität Berlin, Institute of Pharmacy, Königin-Luise-Str. 2-4, 14195 Berlin, Germany

^2^Freie Universität Berlin, Department of Biology, Chemistry, Pharmacy and Physics, SupraFAB, Altensteinstraße 23 a, 14195 Berlin, Germany

Table of Contents

[**Materials** 2](#_Toc197157230)

[Biological materials 2](#_Toc197157231)

[**Methods** 2](#_Toc197157232)

[**Synthesis of antimicrobial polymers** 4](#_Toc197157233)

[Synthesis of precursor polymers 4](#_Toc197157234)

[Post-polymerization functionalization of precursor polymer PPEGMA-*b*-PPFPMA 5](#_Toc197157235)

[**Biological Tests** 7](#_Toc197157236)

[Cytotoxicity Assay 7](#_Toc197157237)

[MIC determination via broth dilution assays 8](#_Toc197157238)

[Synergistic MIC assays 9](#_Toc197157239)

[Hemolysis Assay 10](#_Toc197157240)

[**Micelle formation and CMC determination** 10](#_Toc197157241)

[**Characterization of antimicrobial block copolymers** 12](#_Toc197157242)

[GPC and NMR analysis for synthesis of PPEGMA homopolymer and PPEGMA-*b*-PPFPMA block copolymer 12](#_Toc197157243)

[GPC and NMR analysis for 1^st^ functionalization of PPEGMA-*b*-PPFPMA to PPEGMA-*b*-PMTEMAA 15](#_Toc197157244)

[GPC and NMR analysis for 2^nd^ functionalization of PPEGMA-*b*-PPFPMA to PPEGMA-*b*-PS^+^-X 17](#_Toc197157245)

[GPC analysis for micellization of cationic sulfonium polymers, PPEGMA-*b*-PS^+^-X 24](#_Toc197157246)

[**References** 27](#_Toc197157247)

**Materials**

All starting materials and chemical reagents were purchased from commercial vendors and used as received, except where noted otherwise. 2-(methylthio)ethylamine was purchased from TCI. Methacryloyl chloride (97%), triethylamine (99%) and trifluoroacetic acid (TFA), were obtained from abcr GmbH. Pentafluorophenol, poly(ethylene glycol) methacrylate (PEGMA), (2,3-epoxypropyl)benzene and iodomethane were purchased from Sigma-Aldrich. Ciprofloxacin was purchased from TCI and Penicillin G was purchased from Jenpharm. Dialysis was performed in benzoylated cellulose dialysis tubes from Sigma-Aldrich (width: 32 mm, MWCO: 3-5 kDa).
4-cyano-4-[(ethylsulfanylthiocarbonyl) sulfanyl]pentanoic acid, as CTA, was used as synthesized in Klinger lab. Initiator ACVA, i.e., 4,4'-Azobis(4-cyanovaleric acid) and Pur-A-Lyzer Maxi 6000 dialysis tubes (MWCO, 6−8 kDa) were purchased from Sigma-Aldrich. Sterile, biopure combitips (5 mL) were purchased from Eppendorf and used for broth dilution assays.

**Biological materials**

Defibrinated sheep blood was purchased from Thermo Scientific and used for hemolysis assays. Luria–Bertani (LB) medium (Carl Roth, Art.:X969.1), PBS buffer was purchased from sigma Aldrich. *E. coli* (BL21) strain was used as provided by the group of Prof. J. Rademann (Institute of Pharmacy, FU Berlin). Staphylococcus aureus (ATCC 29213) was used as provided from the group of Prof. C. Kloft (Institute of Pharmacy, FU berlin). L929 fibroblasts (DSMZ no: ACC2) and HaCat keratinocytes (DSMZ no: ACC91) were obtained from Leibniz Institute DSMZ – German Collection of Microorganism and used for cell viability assays. L-Glutamine, Penicillin-Streptomycin and Fetal Bovine Serum (FBS) were obtained from Gibco BRL, Eggenstein, Germany, which were also used for cell viability assays. CCK-8 cytotoxicity assay kit was purchased from Hycultec (Beutelsbach, Germany).

# **Methods**

**Nuclear Magnetic Resonance (NMR) Spectroscopy**

All NMR spectra, including ¹H, ¹⁹F, and ¹³C, were acquired at 300 K using either a Jeol Eclipse 600 MHz spectrometer (Tokyo, Japan) or a Bruker AVANCE 600 MHz spectrometer (Billerica, MA, USA). Chemical shifts (δ) were reported in parts per million (ppm), with deuterated solvent peaks serving as the reference standard. Data processing was performed using MestReNova software (version 6.2.1-7569).

**Gel Permeation Chromatography (GPC)**

**GPC in DMF**

Molecular weight distributions of PPEGMA-CTA, PPEGMA-*b*-PPFPMA precursor, and PPEGMA‑*b*‑PMTEMAA were analyzed using a customized chromatography system provided by PSS Polymer Standards Services GmbH in Mainz, Germany. The mobile phase, composed of DMF containing 10 mmol lithium bromide, was run at a flow rate of 1.0 mL min⁻¹. The columns were maintained at 50 °C. For sample preparation, 1.5 mg of each polymer was dissolved in
1 mL of the mobile phase solvent (DMF with 10 mM LiBr) and filtered through a PTFE (450 μm) filter. A 50 μL aliquot of the filtered sample solution was injected for each analysis. Data processing was performed using PSS WinGPC UniChrom software. Molecular weights and molecular-weight distributions were determined using polystyrene (PS) or polymethylmethacrylate (PMMA) standards from PSS, Mainz, Germany.

**GPC in H_2_O**

The molecular weight distributions of cationic PPEGMA-*b*-PS^+^-me_x_-bz_y_ polymers were analyzed in water by using an Agilent GPC system, which included an Agilent 1100 pump and a refractive index detector. Cationic PLgel columns were employed, with a mobile phase consisting of water and 0.3 M formic acid, flowing at a rate of 1.0 mL min⁻¹. Polyvinylpyrrolidone (PVP) standards were used for calibration. The polymer samples were prepared at a concentration of 3 mg mL⁻¹ and 1.0 mg mL⁻¹ for analysis.

**Dynamic Light Scattering (DLS)**

Micelle formation of BCP-Pen G combinations was examined by monitoring the concentration-dependent scattering intensity at 90°. For this, an aqueous solution of BCP and Pen G (1:1 ratio of Pen G to cationic groups in the BCP) was placed in a multiangle round cell glass cuvette and a NICOMP nano Z3000 (Particle Sizing Systems, USA) was used to determine scattering intensity. For each polymer, the polymer concentration was varied by keeping the molar ratio of Pen G to cations constant. Tests started with the highest concentration of polymer and Pen G.

**Microplate Reader Spectrophotometer**

Optical densities (OD) from the broth dilution assay were measured as absorbance at a wavelength of 600 nm using a Spark 10M instrument (Tecan, Männedorf, Switzerland). The measurements were conducted in sterile, transparent, round bottom 96-well polystyrene microplates purchased from Sarstedt.

# **Synthesis of antimicrobial polymers**

## **Synthesis of precursor polymers**

**Synthesis of poly(polyethylene glycol methacrylate) (PPEGMA)**

The PPEGMA homopolymer was synthesized as macro-CTA. For this, we used a modified literature procedure.^[1]^ In brief, a mixture of PEGMA (10 g, 20 mmol, 52 eq.), 4-cyano-4-[(ethylsulfanylthiocarbonyl) sulfanyl]pentanoic acid (CTA) (106 mg, 383 µmol, 1.0 eq.), and ACVA (12 mg, 42 µmol, 0.125 eq.) was dissolved in 20 mL of anhydrous dioxane. Anhydrous DMF (1 mL) was added as an internal standard. The reaction mixture was purged with nitrogen for 30 minutes, after which the flask was sealed and placed in a pre-heated oil bath at 65 °C for 16 hours. The conversion of the reaction was determined by ^1^H-NMR spectroscopy, comparing the reaction mixture before and after the reaction. The decrease in the integral of the vinyl peaks relative to the DMF peak indicated a monomer conversion of 82%. The resulting polymer was precipitated in cold hexane, centrifuged, redissolved in DCM, and reprecipitated in cold hexane twice. The polymer was then dried under high vacuum, yielding 8.4 g of a yellow oil. A monomer conversion of 82% corresponds to 43 repat units of PEGMA, resulting in a polymer with a molecular weight of 21.5 kDa.

GPC: *M_n_* = 26 000 Da, *M_w_* = 31 000 Da, *Ð* = 1.18

^1^H-NMR (500 MHz, CDCl_3_): δ = 4.07 (s, -COOCH_2_-), 3.76-3.46 (m, -CH_2_(CH_2_)_2_O-),
3.37 (s, -OCH_3_), 2.13-0.66 (m, backbone -CH_2_-, -CH_3_) ppm.

**Synthesis of PPEGMA-*b*-PPFPMA**

The PPEGMA-*b*-PPFPMA block copolymer was synthesized using a modified literature procedure.^[1]^ A mixture of PPEGMA macro-CTA (5 g, 227 µmol, 1.0 eq.), PFPMA (6.86 g, 27.24 mmol, 120 eq.), and ACVA (7.96 mg, 28.4 µmol, 0.125 eq.) was dissolved in 25 mL of anhydrous dioxane, with 1 mL of anhydrous DMF added as an internal standard. The reaction vessel was purged with nitrogen for 30 minutes, sealed, and heated in an oil bath at
80 °C for 16 hours. The conversion of the reaction was determined by ^1^H-NMR spectroscopy, comparing the reaction mixture before and after the reaction. The decrease in the integral of the vinyl peaks relative to the DMF peak indicated an initial monomer conversion of only 23%. To improve the conversion, the reaction was restarted twice, with each cycle involving 30 minutes of nitrogen purging. After the first restart, the reaction mixture was run for additional 72 h and after the second restart, it was run for another 48 h, ultimately achieving a conversion of 76%. The resulting polymer was precipitated in cold hexane, centrifuged, redissolved in DCM, and reprecipitated in hexane twice. The polymer was then dried under high vacuum, yielding 11.3 g of a yellow solid. The 76% monomer conversion corresponds to a PPFPMA block with a molecular weight of 21 kDa (83 PFPMA units).

GPC: *M_n_* = 31 000 Da, *M_w_* = 42 000 Da, *Ð* = 1.24

^1^H-NMR (500 MHz, CDCl_3_): δ = 4.07 (s, -COOCH_2_-), 3.86-3.50 (m, 2H-CH_2_(CH_2_)_2_O-),
3.38 (s, -OCH_3_), 2.65-0.65 (m, backbone -CH_2_-, -CH_3_) ppm.

^19^F-NMR (500 MHz, CD_3_CN): δ = -161.99 (s, J = 16.94 Hz, J = 22.02 Hz, 2F), -156.82 (s, J = 21.65 Hz, 1F), -149.35-152.22 (m, J = 17.80 Hz, 2F) ppm.

## **Post-polymerization functionalization of precursor polymer PPEGMA-*b*-PPFPMA**

1^st^ functionalization of PFPMA units to thioether side group

PPEGMA-*b*-PPFPMA was functionalized using our previously established protocol.^[2]^ Specifically, PPEGMA-*b*-PFPMA (4.8 g, approx. 1.1x10^-4^ mol, corresponding to 9.3 mmol of PFPMA units), was dissolved in 100 ml of DMF and heated to 50 °C. To this solution,
2-(methylthio)ethylamine (4.22 g, 0.046 moles, 5.0 mole eq w.r.t. PFPMA units) was added, followed by the addition of TEA (4.66 g, 0.046 moles, 5.0 mole eq) and stirred for 5 days. Afterwards, the functionalized polymer was purified by dialysis against DMF for 3 days and then against Milli-Q water for 3 further days. The product was lyophilized to yield a brown sticky,
semi-rigid material. Quantitative functionalization was confirmed by ^1^H- and ^19^F-NMR. Yield: 67%.

^1^H-NMR (500 MHz, CDCl_3_): δ = 4.27-3.95 (s, -COOCH_2_-), 3.43-3.30 (m, 2H-CH_2_(CH_2_)_2_O-), 2.78-2.44 S-CH_2,_ NHOC-CH_2_ and S-CH_3_), 2.23-1.99 (m, backbone -CH_2_-, -CH_3_) ppm.

**2^nd^ Functionalization to sulfonium polymers**

The thioether polymer acts as a platform for secondary functionalization. In this step, benzyl epoxide and methyl iodide were employed to simultaneously introduce sulfonium cations as side groups and control hydrophobicity by varying the ratio of methyl (*me*) and benzyl (*bz*) groups.

For the synthesis of PPEGMA-*b*-PS^+^-bz_100_, a previously established protocol was followed. ^[2]^ For the remaining polymers in the library, a modified protocol was used. In cases where both benzyl and methyl functional groups were incorporated, (2,3-epoxypropyl)benzene and methyl iodide were added in varying ratios (as detailed in Table S1).

In a representative reaction for synthesizing PPEGMA-*b*-PS^+^-bz_y_*,* the thioether polymer was dissolved in DMF and heated to 50 °C. Methyl iodide (CH_3_I) was added and the mixture was stirred overnight. Subsequently, (2,3-epoxypropyl)benzene and trifluoroacetic acid (TFA) were added, and the reaction was stirred for an additional day. For PPEGMA-*b*-PS^+^-me_100_, the reaction was conducted in DMF at 50 °C using only an excess of CH_3_I (12 equivalents).

After the reaction was ended, the functionalized polymer was dialyzed against DMF for 2 days followed by dialysis against Mili-Q water for another day. Moreover, to exchange the TFA and iodide counter anions with chloride anions, all polymers were extensively dialyzed against 0.1 M NaCl solution for 5 days. Ultimately, the samples were dialyzed against Mili-Q water to remove excess NaCl. The purified polymers were then freeze dried and characterized through ^1^H-NMR spectroscopy.

PPEGMA-*b*-PS^+^-me_100_. ^1^H-NMR (500 MHz, D_2_O): δ = 4.43-3.17 (m, -CH_2_(CH_2_)_2_O- and S-CH_2_ and NHOC-CH_2_), 2.84-3.13 (br s, S-(CH_3_)_2_), 2.37-0.58 (m, backbone -CH_2_-, -CH_3_) ppm.

PPEGMA-*b*-PS^+^-bz_30_. ^1^H-NMR (500 MHz, D_2_O): δ = 7.43-7.17 (s, Ar-CH), 4.44-4.03 (m, CH_2_(CH_2_)_2_O- and S-CH_2_ and NHOC-CH_2_), 3.89-3.17 (br s, S-(CH_3_)_2_), 3.08-2.67 (m, backbone -CH_2_-, -CH_3_) ppm.

PPEGMA-*b*-PS^+^-bz_50_. ^1^H-NMR (500 MHz, D_2_O): δ = 7.48-7.11 (s, Ar-CH), 4.44-3.14 (m, CH_2_(CH_2_)_2_O- and S-CH_2_ and NHOC-CH_2_), 3.09-2.74 (br s, S-(CH_3_)_2_), 2.32-0.54 (m, backbone
-CH_2_-, -CH_3_) ppm.

PPEGMA-*b*-PS^+^-bz_70_. ^1^H-NMR (500 MHz, D_2_O): δ = 7.51-7.19 (s, Ar-CH), 4.48-4.04 (m, CH_2_(CH_2_)_2_O- and S-CH_2_ and NHOC-CH_2_), 3.11-2.77 (br s, S-(CH_3_)_2_), 2.39-0.75 (m, backbone -CH_2_-, -CH_3_) ppm.

PPEGMA-*b*-PS^+^-bz_80_. ^1^H-NMR (500 MHz, D_2_O): δ = 7.43-6.89 (s, Ar-CH), 4.38-3.04 (m, CH_2_(CH_2_)_2_O- and -S-CH_2_ and NHOC-CH_2_), 3.02-2.66 (br s, S-(CH_3_)_2_), 2.2-0.53 (m, backbone,
-CH_2_-, -CH_3_) ppm.

PPEGMA-*b*-PS^+^-bz_100_. ^1^H-NMR (500 MHz, D_2_O): δ = 7.65-7.04 (s, Ar-CH), 4.52-3.2
(m, 2H-CH_2_(CH_2_)_2_O- and S-CH_2_ and NHOC-CH_2_), 3.08-2.82 (br s, S-(CH_3_)_2_), 2.44-0.74 (m, backbone, -CH_2_-, -CH_3_) ppm.

**Table S1:** Synthesis of cationic BCPs with varying bz:me ratios: Amounts of methyl iodide and benzyl epoxide for functionalization of PPEGMA-b-PMTEMAA, amount of polymer was kept constant as 0.5 g for all reactions.

| **Polymer** | **CH_3_I** | | | **benzyl epoxide** | | | **TFA** |
| --- | --- | --- | --- | --- | --- | --- | --- |
|  | eq. | n  [mmol] | m  [µg] | eq. | n  [mmol] | m  [µg] | m  [µg] |
| PPEGMA-*b*-PS^+^-bz_100_ | - | - | - | 6.0 | 7.3 | 975 | 828 |
| PPEGMA-*b*-PS^+^-bz_80_ | 4.5 | 5.4 | 773 | 4.2 | 5.1 | 682 | 579 |
| PPEGMA-*b*-PS^+^-bz_70_ | 6 | 2.9 | 412 | 2.5 | 3.0 | 406 | 345 |
| PPEGMA-*b*-PS^+^-bz_50_ | 9 | 6.5 | 927 | 1.5 | 1.1 | 146 | 124 |
| PPEGMA-*b*-PS^+^-bz_30_ | 10.5 | 3.02 | 429 | 0.9 | 0.43 | 58 | 50 |
| PPEGMA-*b*-PS^+^-me_100_ | 15 | \| 18.2 \| \| --- \| | 272 | - | - | - | - |

# **Biological Tests**

## **Cytotoxicity Assay**

Cell viability assays were conducted on HaCat and L929 cell lines using the Cell Counting Kit-8 (CCK-8) assay. All cell experiments adhered to German genetic engineering laws and biosafety guidelines, performed in a laboratory with a biosafety level of 2. The CCK-8 Kit (Hycultec; Art. HY-K0301) was used to assess cell viability following the manufacturer’s protocol. HaCat cells were maintained in Roswell Park Memorial Institute (RPMI) 1640 Medium, while L929 cells were cultured in Dulbecco’s Modified Eagle Medium (DMEM), both supplemented with 10% (v/v) fetal bovine serum (FBS), 100 U mL^-1^ penicillin, and 100 µg mL^-1^ streptomycin.

Cells were seeded in a 96-well plate at a density of 5 × 10⁴ cells/mL, with 90 µL of RPMI or DMEM medium per well, and incubated overnight at 37 °C with 5% CO₂. Serial dilutions of sample solutions (prepared in sterile deionized water) were added to the wells (10 µL per well), alongside 1% SDS as dead control and culture medium and H₂O were used as non-treated control or living controls, followed by incubation for 24 h under the same conditions. Wells containing only polymer solutions (no cells) were used for background signal subtraction. After incubation, 10 µL of CCK-8 solution was added to each well, and the plates were incubated for an additional 3 h. Absorbance was measured at 450 nm (reference: 650 nm) using a Tecan plate reader (SPARK, Tecan Group Ltd.).

All measurements were performed in triplicate and repeated three times. Cell viability was calculated by normalizing the non-treated control to 100% and the non-cell control to 0%, after subtracting the background signal. Data were analysed using Origin software, with mean viability values (n = 3) and standard deviations plotted for each polymer concentration.

## **MIC determination via broth dilution assays**

All experiments involving bacteria were conducted in compliance with German genetic engineering laws and biosafety guidelines in a safety level 2 laboratory. To determine antimicrobial activity of compounds, we followed a previously established procedure.^[2–4]^ In brief, broth dilution assays were performed in a 96-well microtiter plate to evaluate the susceptibility of *E. coli* (gram-negative) and *S. aureus* (gram-positive) to individual block copolymers (BCPs) and antibiotics as a reference. Precultures were prepared by inoculating glycerol stock solutions in 5 mL of LB medium, followed by incubation over night at 37 °C with shaking at 140 rpm. Optical density (OD_600_) of the bacterial cultures was determined at a wavelength of 600 nm via Tecan plate reader. Then, bacterial cultures were diluted to an OD_600_ of 0.02 for all experiments. Polymer stock solutions (1024 μg mL⁻^1^) were prepared in sterile Milli-Q water and serially diluted by 2-fold dilutions (from 512 to 0.5 μg mL⁻^1^, 100 µL per well in 96-well plates). Subsequently, 100 μL of the diluted bacterial suspension (OD ~ 0.02) was added to each well, resulting in final polymer concentrations of 256, 128, 64, 32, 16, 8, 4, 2, 1, 0.5, and 0.25 μg mL⁻^1^. Similarly, MIC of each antibiotic was determined by the same method as used for BCPs. Positive controls (LB medium with bacteria, no polymer) and negative controls (LB medium without bacteria or polymer) were included. Polymer controls (without bacteria) were also measured to account for any principates or polyplex formation in LB medium. All samples and controls were tested in triplicates and incubated overnight at 37 °C. Inhibition percentages were calculated using the formula:

$$Inhibition \%=\frac{OD600(sample)-OD600(negative control)}{OD600(positive control)-OD600(negative control)} X 100$$

Here, OD_600_(sample) represents the OD_600_ value of bacteria incubated with the polymer, OD_600_ (negative control) represents the OD_600_ value without bacteria, and OD_600_(positive control) represents the OD_600_ value of bacteria without polymer. The MIC_90_ was defined as the lowest polymer concentration that inhibited over 90 % of bacterial growth.

## **Synergistic MIC assays**

To determine the influence of BCPs on the antimicrobial activity of penicillin G and ciprofloxacin, bz_30_ and bz_80_ polymers were selected as representatives of a hydrophilic BCP and a more hydrophobic BCP. Their effect on the activity of the antibiotics was determined against *S. aureus* and *E. coli* via the following method*:* For each strain, a preculture was prepared by inoculating a glycerol stock solution in 5 mL of LB-Medium, which was incubated overnight at 37 °C with shaking at 140 rpm. The OD_600_ of the bacterial culture was measured using absorbance reader and diluted to an OD value of 0.02 before adding to the wells for experiments. For each polymer, 4 mL stock solutions with 8, 16, 32, 64, 128, 256, and 512 μg mL^−1^ were prepared in sterile
Milli-Q water in 5 mL sterile vials. 100 μL of each stock solution were added to a new empty well in a 96-well plate. 500 µL of each polymer stock were used to prepare combined polymer-antibiotic stock solutions: For *E. coli*, 128 μg mL^−1^ and 75 ng mL^−1^ stock solutions of penicillin G and ciprofloxacin were prepared for each polymer concentration, respectively. For *S. aureus*, 16 μg mL^−1^ stock solutions of both penicillin G and ciprofloxacin were prepared for each polymer concentration. The stock solutions of polymer-antibiotic combinations were then used to prepare a series of different concentrations by 2-fold dilutions (each 100 µL in 96-well plates). For the MIC test, 100 μL of the diluted bacterial suspension (OD ~ 0.02) were prepared and added to each well. This gave final concentrations for each antibiotic and polymer according to the dilution of their stock solutions. For instance, for Pen G, each 128 μg mL^−1^ stock solution with a different polymer concentration was diluted to further 11 concentrations i.e., 32, 16, 8, 4, 2, 1, 0.5, 0.25, 0.125, 0.075, 0.0375 μg mL^−1^. This gave combinations of each antibiotic concentration with a respective polymer concentration. For each 75 ng mL^−1^ stock solution of cipro, 18.75, 9.4, 4.68, 2.34, 1.17, 0.586, 0.293, 0.147 ng mL^−1^ concentrations were tested. For each 16 μg mL^−1^ stock solution of both Pen G and cipro, 4, 2, 1, 0.5, 0.25, 0.125, 0.075, 0.0375, 0.0188, 0.0094, 0.0047 µg mL^−1^ concentrations were tested. Positive and negative controls, without antimicrobial agent and bacteria, respectively, were also included. The plates were incubated at 37 °C for 20 h, and the absorbance at 600 nm was subsequently recorded. From here, MICs of antibiotics were calculated in combination with polymer, where polymer and antibiotics combined inhibited more than 90 % of bacterial growth. The MICs of individual polymer and antibiotics were used as calculated from above MIC test. Fractional inhibitory concentration index (FICI) values were calculated from the following equation:^[5]^

$$\mathrm{FICI}=\frac{MICpolym in combination}{MICpolym}+\frac{MICdrug in combination}{MICdrug}$$

FICI helped to determine the interactions between polymer and drugs. The FICI values were interpreted as follows: FICI ≤ 0.5 indicated a synergistic effect, 0.5 < FICI ≤ 1 suggested additive, 1 < FICI ≤ 2 indicated indifference, and >2 represented an antagonistic effect. All experiments were conducted in at least two independent biological replicates.

## **Hemolysis Assay**

Hemolytic activity of the block copolymers was evaluated using fresh sheep erythrocytes. Blood samples were centrifuged at 3500 rpm to remove the serum, and the pelleted erythrocytes were resuspended in an equal volume of PBS buffer (137 mM NaCl, 2.7 mM KCl, 10 mM Na₂HPO₄, 0.24 mM KH₂PO₄, pH 7.4). This washing step for erythrocytes was repeated three times. The assays were carried out following a reported protocol with minor adjustments.^[6]^ Briefly, conical-bottom 96-well microplates were used to conduct this experiment and 100 µL of PBS was added to an empty well. Each polymer was dissolved in PBS to make stock solution of 16384 µg mL^-1^, which was further diluted in 2-fold dilution series to make various concentrations (8192, 4096, 2048, 1024, 512, 256, 128, 64 µg mL^-1^) for each polymer (100 µL per well). Then, 100 µL of erythrocyte suspension (2.5% of total volume assay) was added subsequently to each well, which created the final concentrations (4096, 2048, 1024, 512, 256, 128, 64, 32 µg mL^-1^) of polymers. Positive controls (10% v/v Triton X-100 in PBS) and negative controls (PBS alone) were used to represent 100% and 0% hemolysis, respectively. The microplates were incubated at 37 °C for
1 hour, then centrifuged at 1500 rpm for 10 minutes. The supernatants were transferred to empty wells in a new 96-well plate, and absorbance was measured at 543 nm using Tecan microplate reader. A colorless supernatant indicated no hemolysis, while a red solution indicated hemolysis.

$$Hemolysis \%=\frac{\mathrm{Asample}-Anegative control}{Apositive control-Anegative control} X 100$$

The percentage of hemolysis was calculated using the provided equation. Each experiment was performed in quadruplicate, and the results were expressed as mean ± SD (n = 4). The data are presented as HC_50_, which represents the polymer concentration causing 50 % hemolysis.

# **Micelle formation and CMC determination**

In 1 mL glass vials, polymer solutions in filtered Milli-Q water were prepared at concentrations ranging from 1.25 µg mL^-1^ to 1.5 mg mL^-1^ (1.25, 2.5, 5, 10, 25, 50, 100, 250, 500, 1000, and 1500 µg mL^-1^). The solutions were stirred to ensure homogeneity and left overnight with sealed lids to prevent evaporation. The next day, Pen G was added to each vial at a 1:1 molar ratio relative to the polymer, with all concentrations calculated as outlined in Table S2. The vials were then sealed and stirred continuously for 48 hours. To ensure reproducibility across all polymers, the experimental setup was kept consistent in terms of using the same vials, stir bars, stirring speed, and stirring plate. After incubation, scattering intensity for all samples was determined via dynamic light scattering (DLS) (see Methods section) and plotted against the polymer concentration. Each plot showed two regions that each were fitted via linear regression. From these plots, the critical micelle concentration (CMC) was determined as the x-value of the intersection of both linear fits. Micelle formation occurred at different concentrations for each polymer, depending on their benzyl content, and was visually confirmed by a color change from transparent to violet. This approach ensured consistent and reproducible results across all experiments.

Table S2. Amounts of polymer and Pen G used for micelles formation.

| amount of polymer [mg] | amount of Penicillin G [mg] | | | | |
| --- | --- | --- | --- | --- | --- |
| Polymer | bz_100_ | bz_80_ | bz_70_ | bz_50_ | bz_30_ |
| 0.125 | 0.071 | 0.072 | 0.068 | 0.072 | 0.068 |
| 0.25 | 0.142 | 0. 143 | 0.135 | 0.143 | 0.135 |
| 0.333 | 0.188 | 0.191 | 0.180 | 0.191 | 0.180 |
| 0.416 | 0.235 | 0.238 | 0.225 | 0.238 | 0.225 |
| 0.499 | 0.283 | 0.286 | 0.270 | 0.286 | 0.270 |
| 0.582 | 0.329 | 0.333 | 0.315 | 0.333 | 0.315 |
| 0.665 | 0.376 | 0.381 | 0.360 | 0.381 | 0.360 |
| 0.748 | 0.423 | 0.429 | 0.405 | 0.429 | 0.405 |
| 0.831 | 0.470 | 0.476 | 0.450 | 0.476 | 0.450 |
| 0.914 | 0.517 | 0.524 | 0.494 | 0.524 | 0.494 |
| 0.997 | 0.566 | 0.571 | 0.539 | 0.571 | 0.539 |
| 1.25 | 0.708 | 0.716 | 0.676 | 0.716 | 0.676 |
| 1.5 | 0.849 | 0.860 | 0.812 | 0.860 | 0.812 |

# **Characterization of antimicrobial block copolymers**

## **GPC and NMR analysis for synthesis of PPEGMA homopolymer and PPEGMA-*b*-PPFPMA block copolymer**

GPC traces of PPEGMA and PPEGMA-*b*-PPFPMA


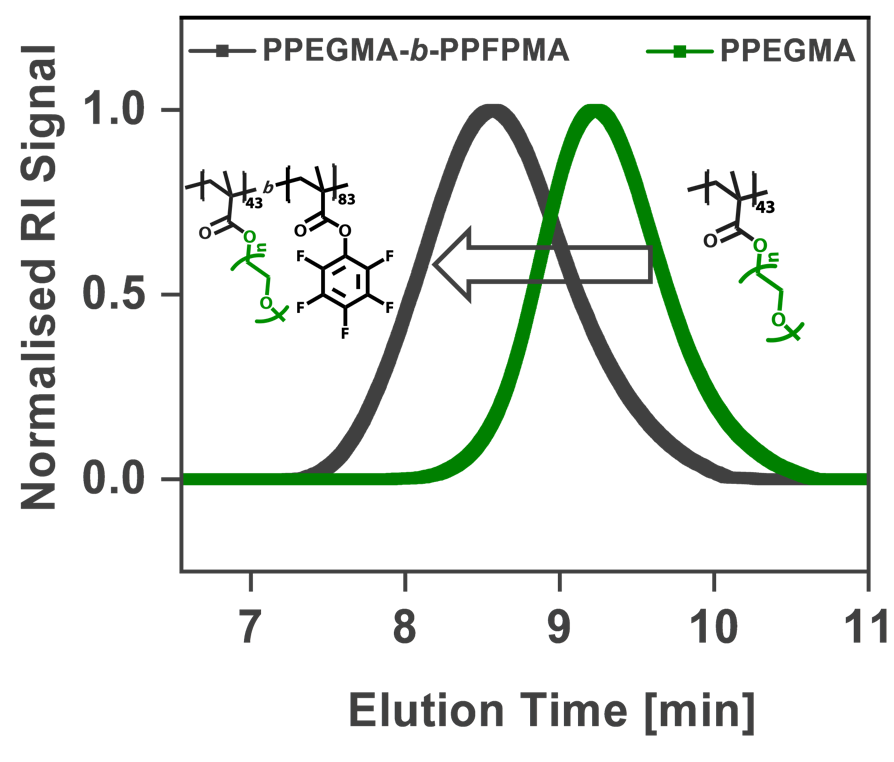


**Figure S1.** GPC traces of PPEGMA vs. PPEGMA-*b*-PPFPMA. A shift to lower elution times after block copolymerization suggests the successful formation of a diblock copolymer.

^1^H-NMR analysis of PPEGMA homopolymer and PPEGMA-*b*-PPFPMA block copolymer

Since different solubility behavior of both blocks can lead to inaccurate molecular weight determination via GPC, molecular weight was also determined via ^1^H-NMR spectroscopy. For this, a constant amount of DMF was added as an internal standard in the RAFT polymerization reaction. Integrals of vinyl proton peaks before and after polymerization were then compared to the integral of the DMF protons. This enabled to determine the conversion of the polymerization, which can be translated into the degree of polymerization and the corresponding molecular weight.


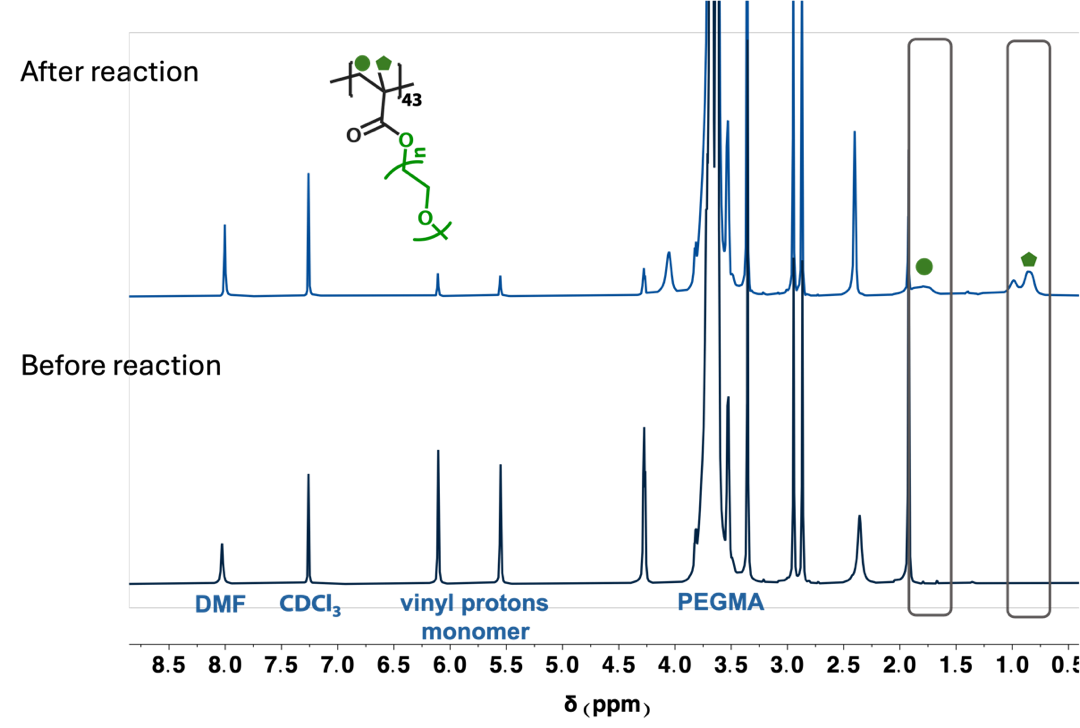


**Figure S2.** ^1^H NMR analysis indicates an 83 % conversion for the polymerization of PEGMA. This was determined by comparing the integrals of the vinyl protons in the monomer before and after the synthesis, using DMF as an internal standard. The resulting product corresponds to a
21.5 kDa PPEGMA_500_ block.


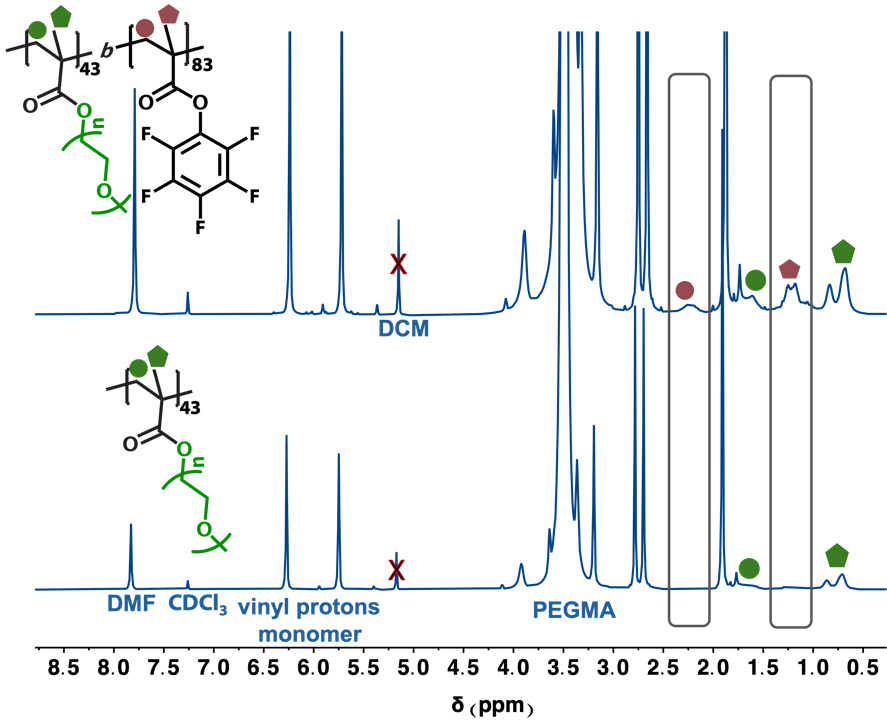


**Figure S3.** ^1^H-NMR analysis confirms the successful formation of the PPEGMA-*b*-PPFPMA as evidenced by proton signals at 1.25 and 2.25 ppm. The spectrum indicates a 76 % conversion for the polymerization of PFPMA initiated by the PPEGMA macro-RAFT agent. Conversion was determined by comparing the integrals of the monomer's vinyl protons before and after synthesis, using DMF as an internal standard, yielding a 22 kDa PPFPMA block.

## **GPC and NMR analysis for 1^st^ functionalization of PPEGMA-*b*-PPFPMA to PPEGMA-*b*-PMTEMAA**


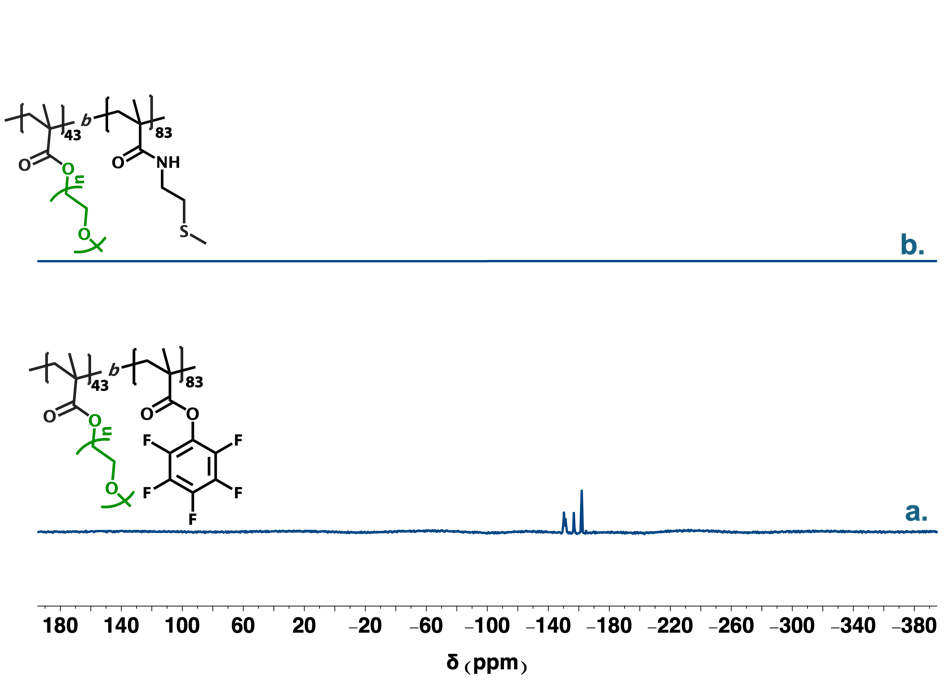


**Figure S4:** ^19^F-NMR analysis suggests quantitative functionalization of PPFPMA block to PMTEMAA block due to disappearance of fluorine signals after functionalization. a) ^19^F NMR of PPEGMA-*b*-PPFPMA and b) ^19^F NMR of PPEGMA-*b*-PMTEMAA.

**
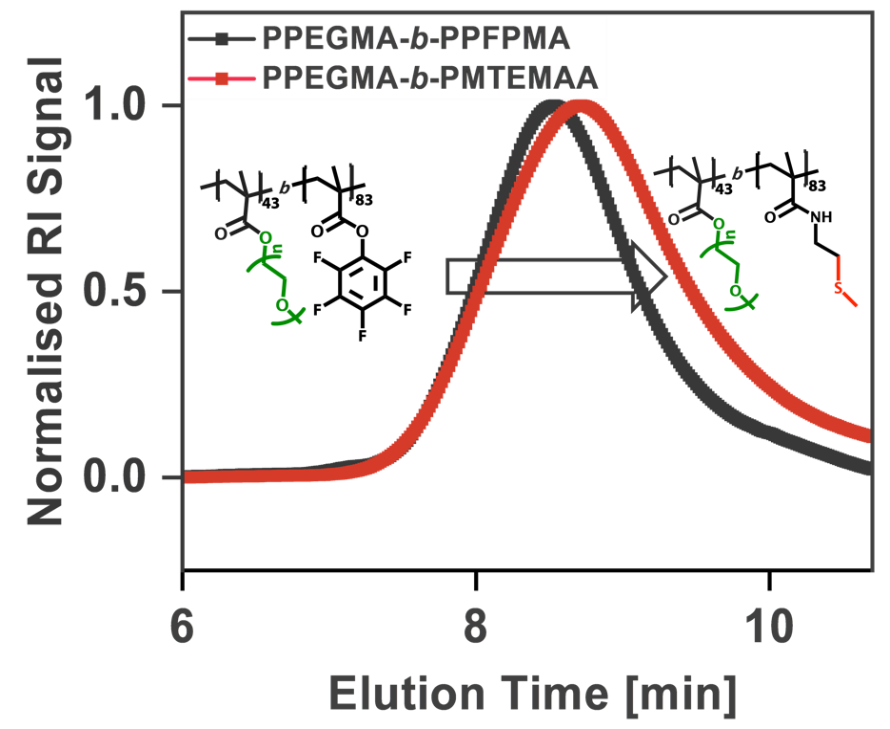
**

**Figure S5:** GPC analysis for 1^st^ functionalization of PPEGMA-*b*-PPFPMA to
PPEGMA-*b*-PMTEMAA block copolymer.

**
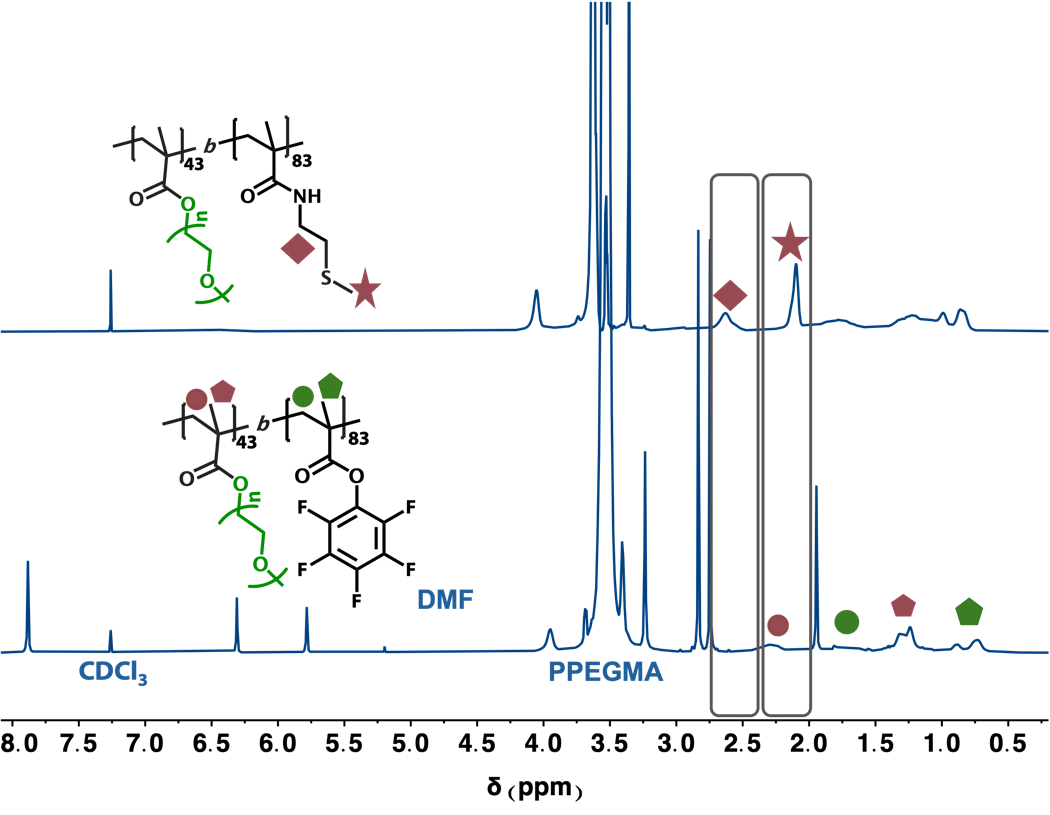
**

**Figure S6.** The successful functionalization of PFPMA units to the thioether units in PPEGMA‑*b*‑PMTEMAA, is confirmed by comparing ^1^H-NMR spectra of PPEGMA-*b*-PPFPMA and PPEGMA-*b*-PMTEMAA. The appearance of methyl and methylene peaks from methylthioethyleneamine (MTEA) in PPEGMA-*b*-PMTEMAA confirms the successful functionalization.

## **GPC and NMR analysis for 2^nd^ functionalization of PPEGMA-*b*-PPFPMA to PPEGMA-*b*-PS^+^-X**


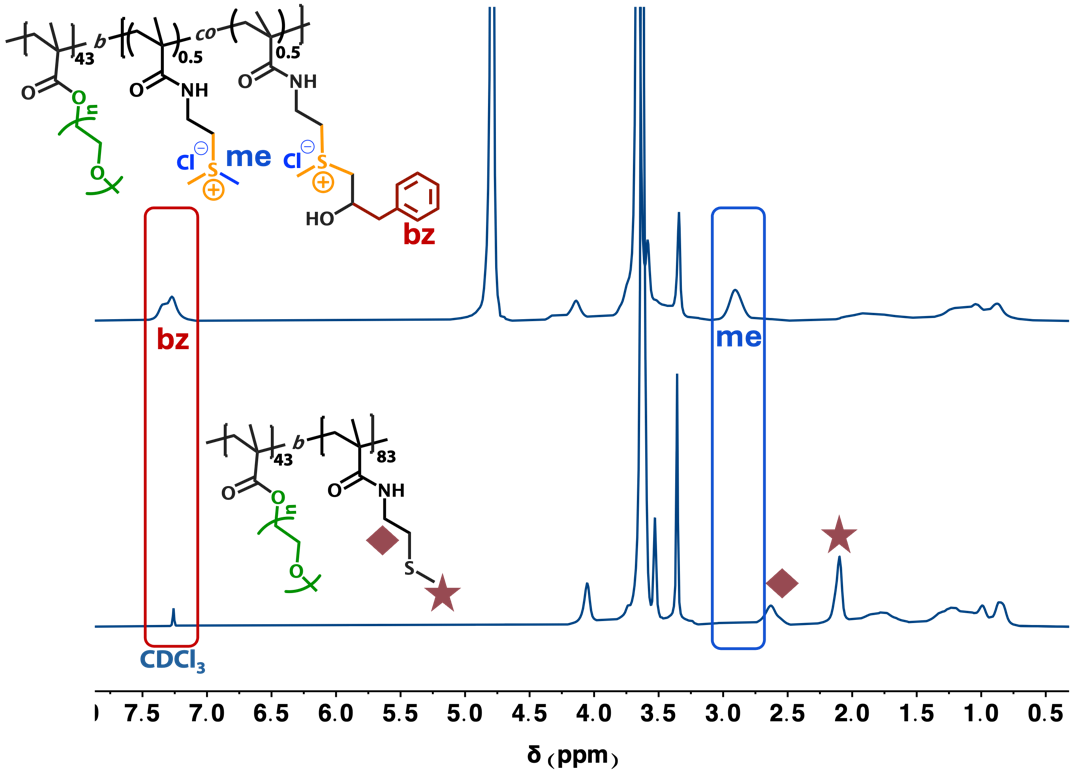


**Figure S7.** The successful functionalization of MTEMAA units to sulfonium groups is confirmed by comparing ^1^H-NMR spectra of PPEGMA-*b*-PMTEMAA and PPEGMA-*b*-PS^+^-bz_0.5_. The appearance of methyl peaks at 3.0 ppm and aromatic proton peaks around 7.3 ppm confirms the successful functionalization of thioether units to sulfonium cations with benzyl and methyl side groups.

**
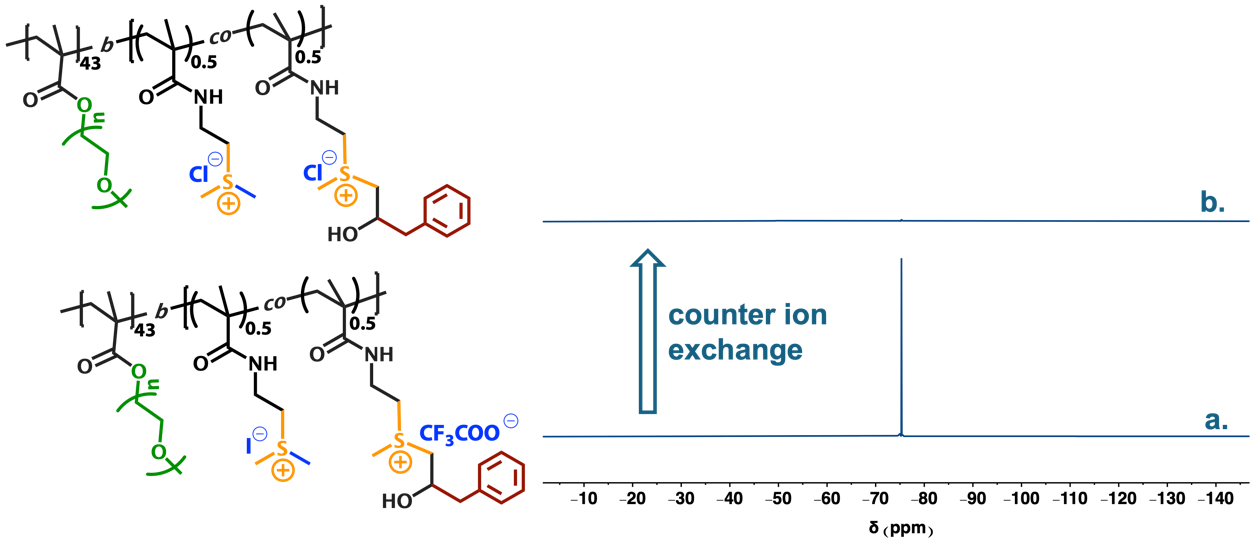
**

**Figure S8.** ^19^F-NMR analysis for counter ion exchange a) before and b) after dialysis with NaCl. The disappearance of fluorine signals after dialysis suggests successful exchange of the CF_3_COO^-^ anions.

**
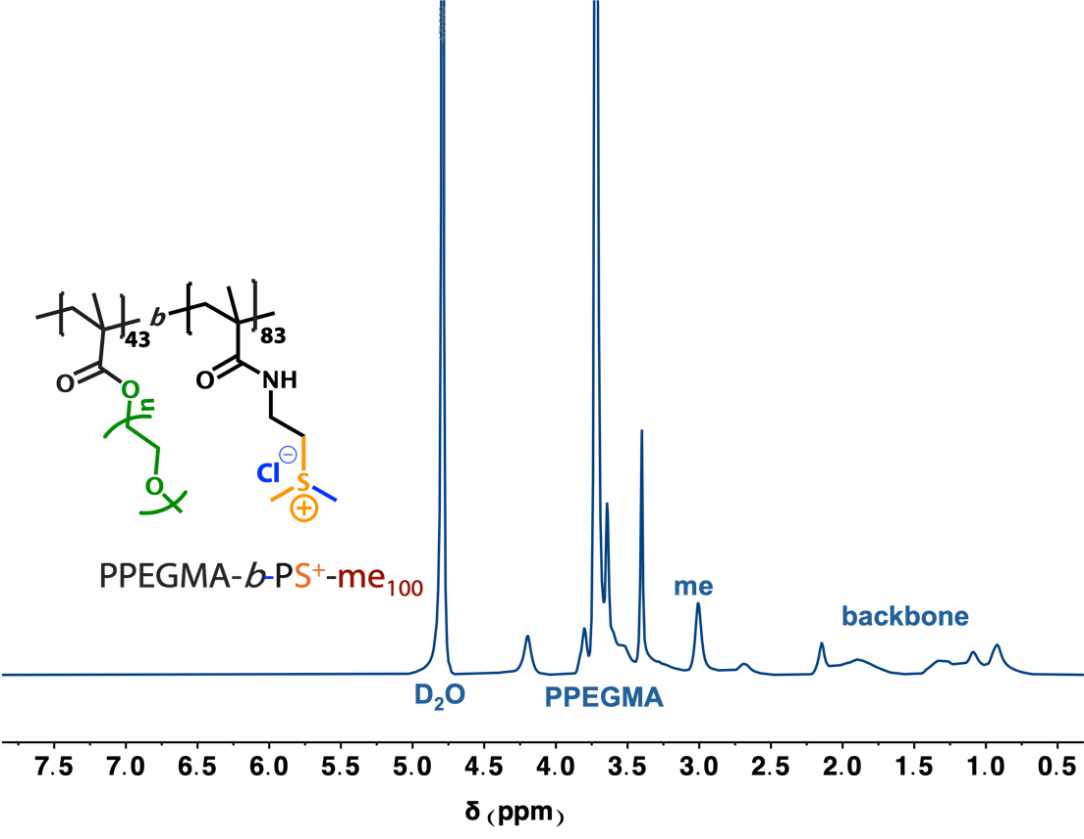
**

**Figure S9.** ^1^H-NMR spectrum of PPEGMA-*b*-PS^+^-me_100_.

**
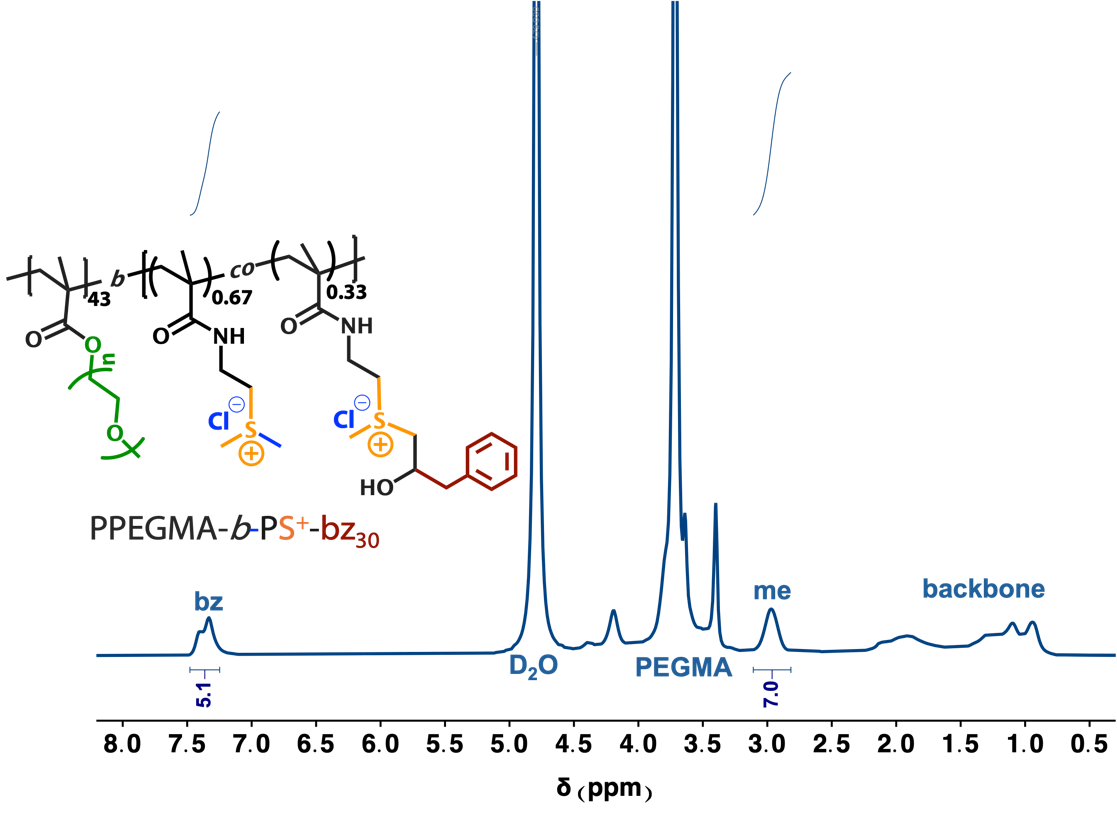
**

**Figure S10.** ^1^H-NMR spectrum of PPEGMA-*b*-PS^+^-bz_30._

**
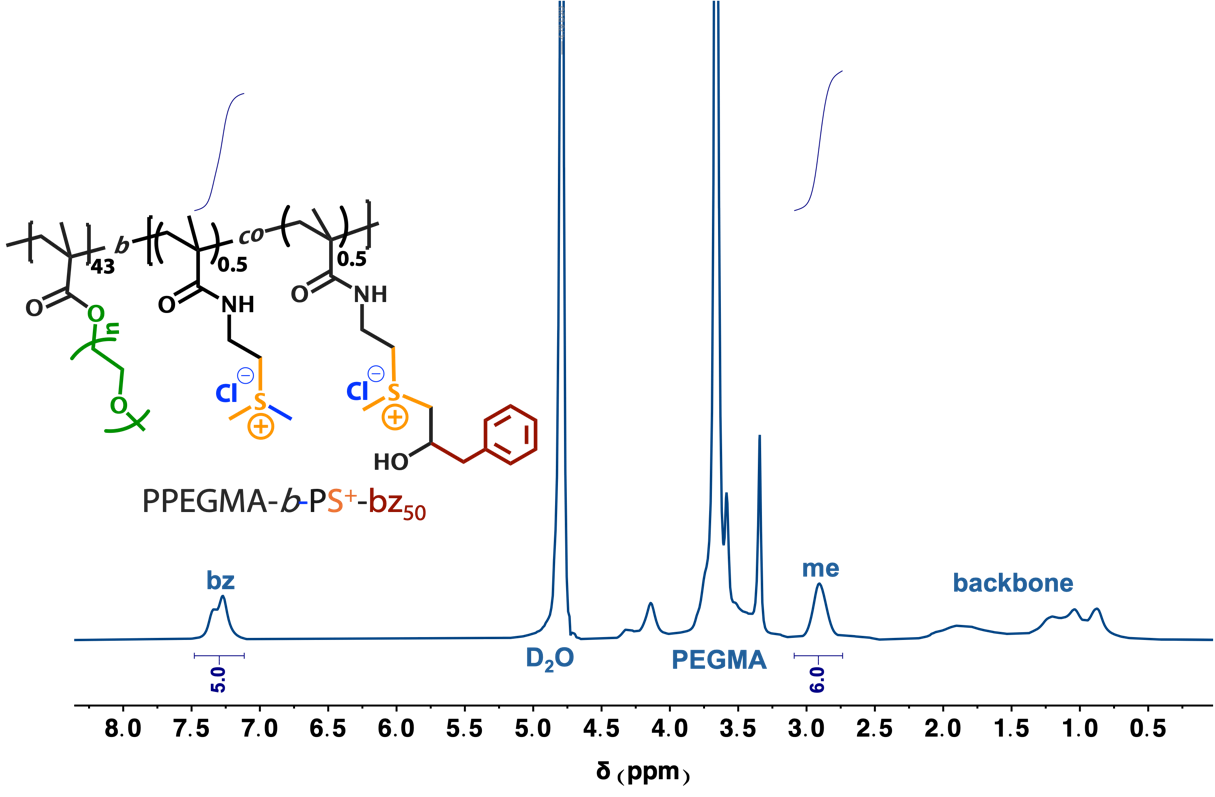
**

**Figure S11.** ^1^H-NMR spectrum of PPEGMA-*b*-PS^+^-bz_50_.

**
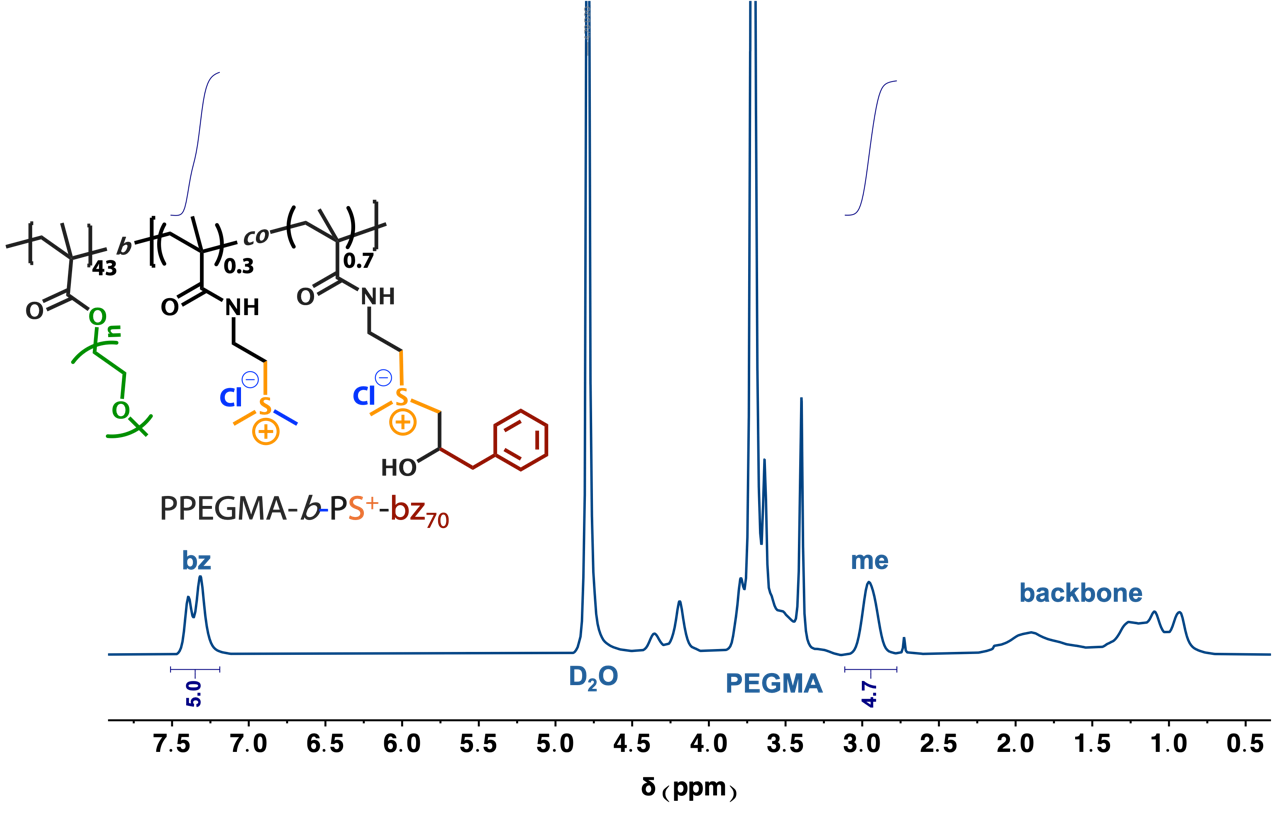
**

**Figure S12.** ^1^H-NMR spectrum of PPEGMA-*b*-PS^+^-bz_70_.

**
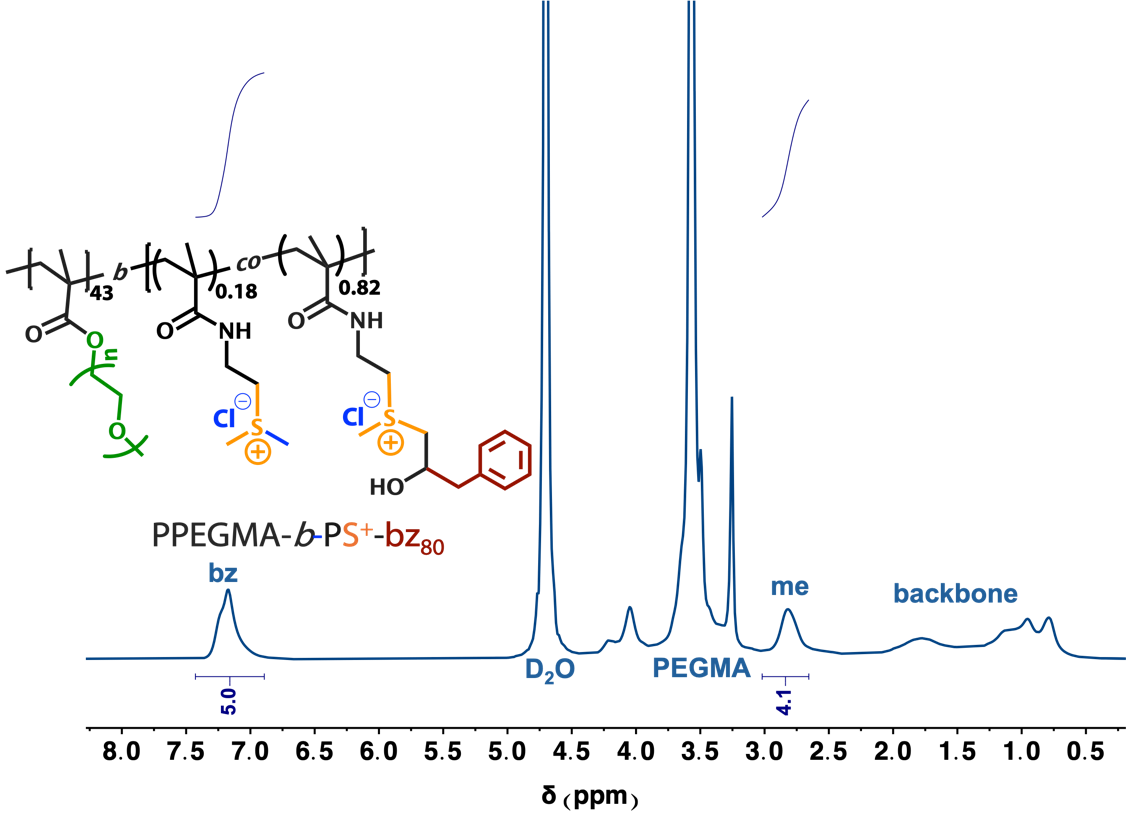
**

**Figure S13.** ^1^H-NMR spectrum of PPEGMA-*b*-PS^+^-bz_80_.

**
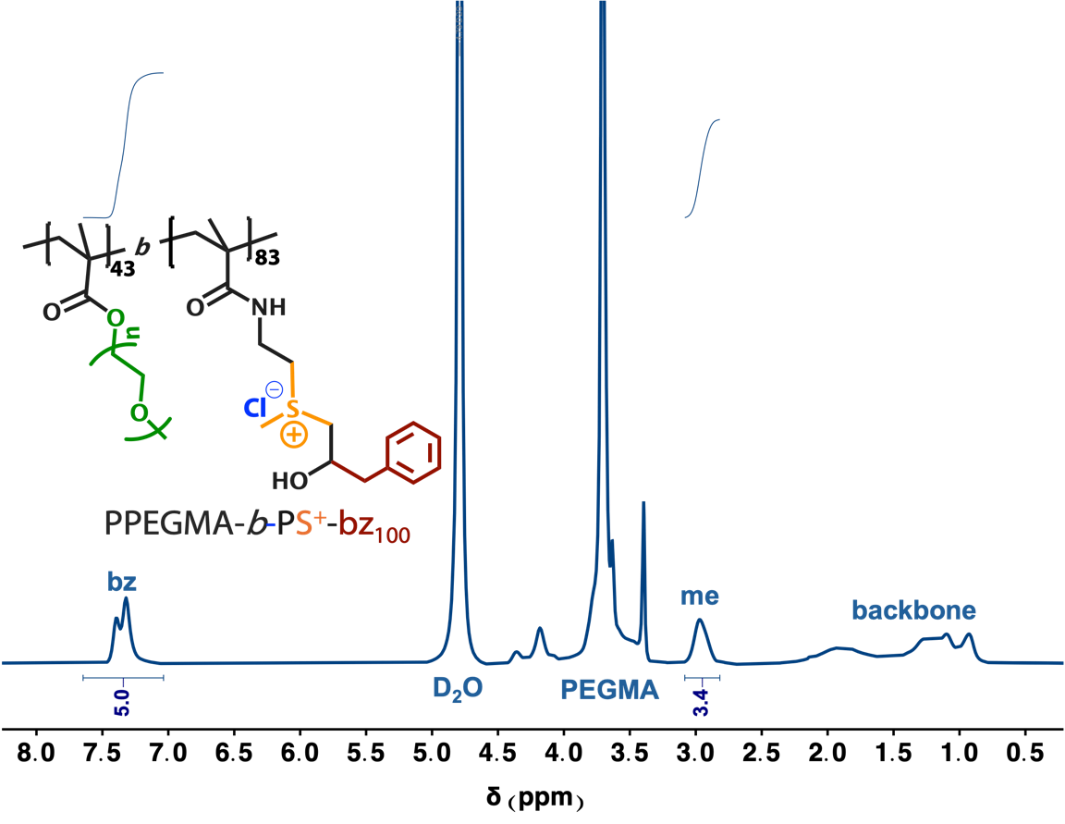
**

**Figure S14.** ^1^H-NMR spectrum of PPEGMA-*b*-PS^+^-bz_100._

**Table S3.** Experimentally determined degree of functionalization of sulfonium block copolymers with methyl (me) and benzyl (bz) groups.

| **Polymer** | **degree of functionalization [%]** | |
| --- | --- | --- |
|  | **me** | **bz** |
| PPEGMA-*b*-PS^+^-bz_100_ | >99.9 | - |
| PPEGMA-*b*-PS^+^-bz_80_ | 18 | 82 |
| PPEGMA-*b*-PS^+^-bz_70_ | 28 | 72 |
| PPEGMA-*b*-PS^+^-bz_50_ | 50 | 50 |
| PPEGMA-*b*-PS^+^-bz_30_ | 33 | 66.6 |
| PPEGMA-*b*-PS^+^-me_100_ | - | >99.9 |


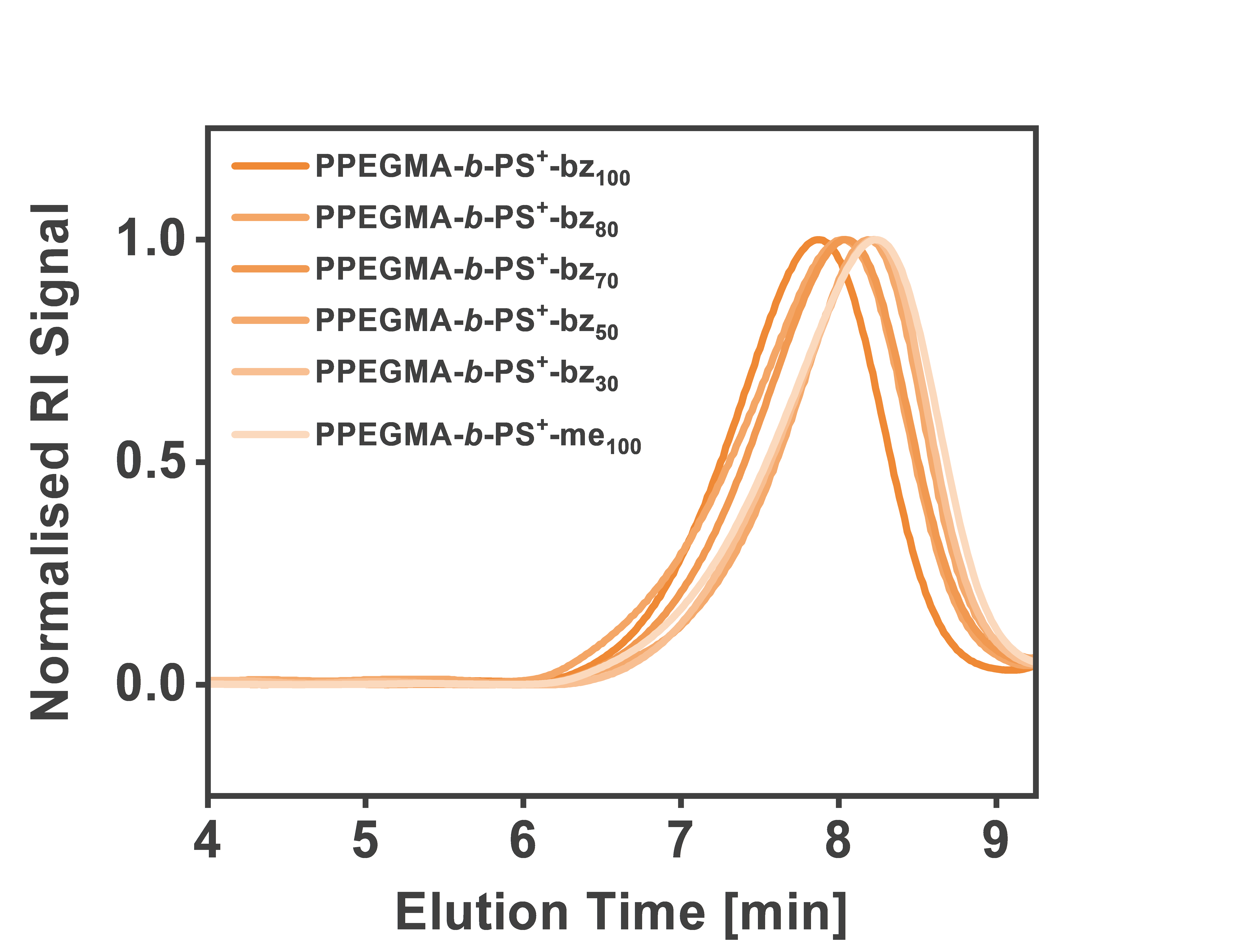


**Figure S15.** GPC traces of cationic sulfonium block copolymers. The difference in their elution times demonstrate their different molecular weights but similar distribution.

**Cytotoxicity of sulfonium block copolymers on L929 cells**

**
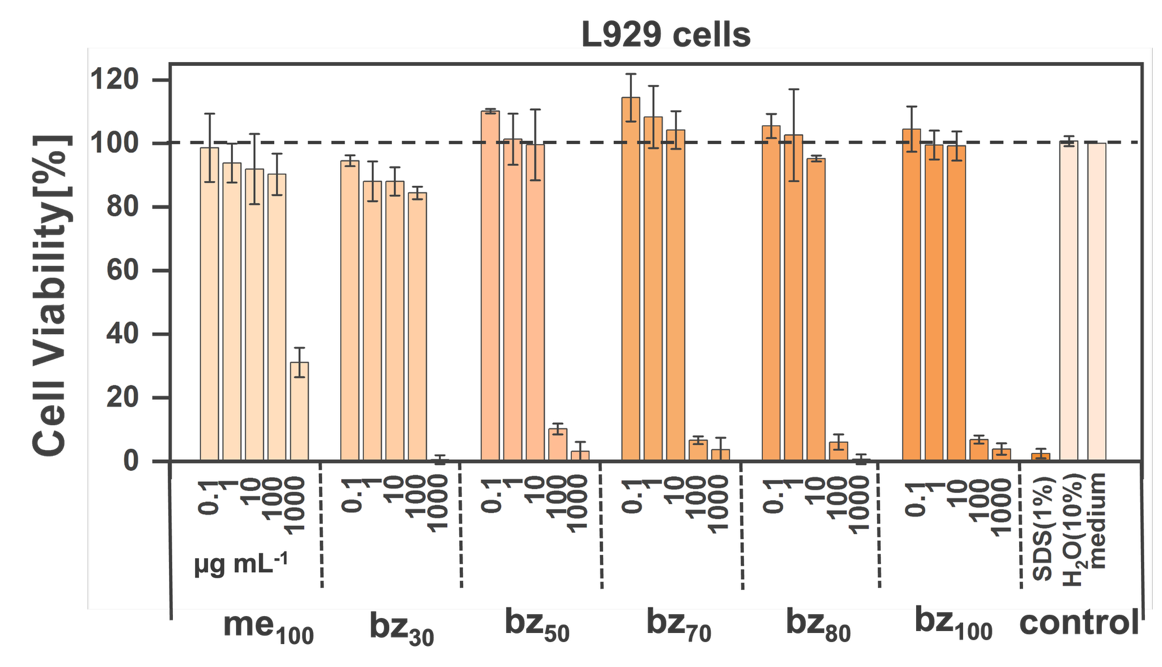
**

**Figure S16.** Cell viability assays on mouse dermal fibroblast cells (L929) demonstrate no significant cytotoxic effects at polymer concentrations below 100 µg mL^-1^, indicating the cellular compatibility of the functional polymers at these levels. However, reduced cell viability was observed at higher concentrations. Cell viability, expressed as a percentage relative to the medium, is presented as mean ± SD (n = 3). The data were obtained using the CCK-8 assay after 24-hour incubation of cells with aqueous solutions of the hydrophobic sulfonium polymers at concentrations of 0.1, 1, 10, 100, and 1000 µg mL^−1^.

Chemical structures of antibiotics


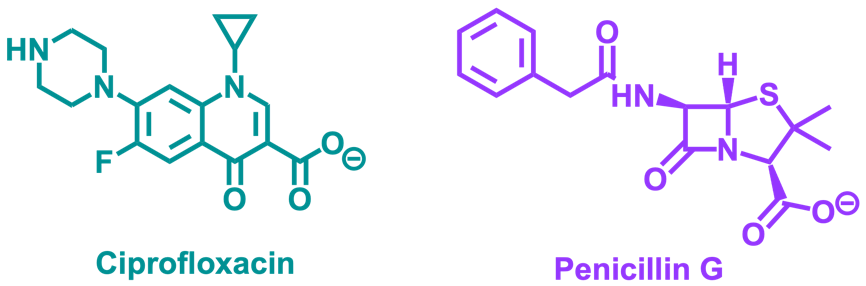


Figure S17. Chemical structures of ciprofloxacin and Penicillin G.

## **GPC analysis for micellization of cationic sulfonium polymers, PPEGMA-*b*-PS^+^-X**

**
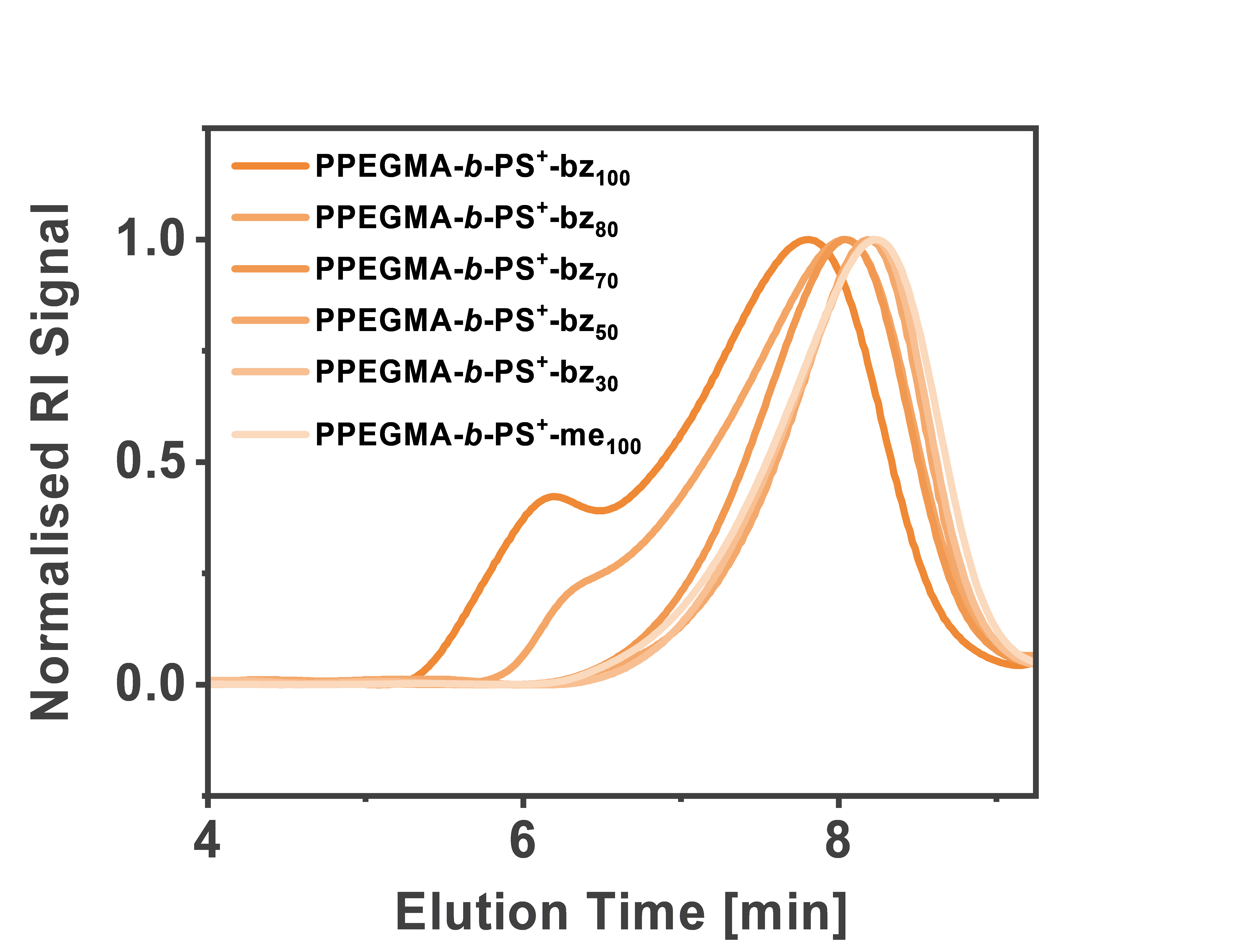
**

**Figure S18.** GPC traces of cationic sulfonium block copolymers in water. For PPEGMA‑b‑PS^+^‑bz_80_ and PPEGMA-b-PS^+^-bz_100_, the shoulders at lower elution times suggest micelle formation without antibiotics.

**DLS study for micelles formation and CMC determination**


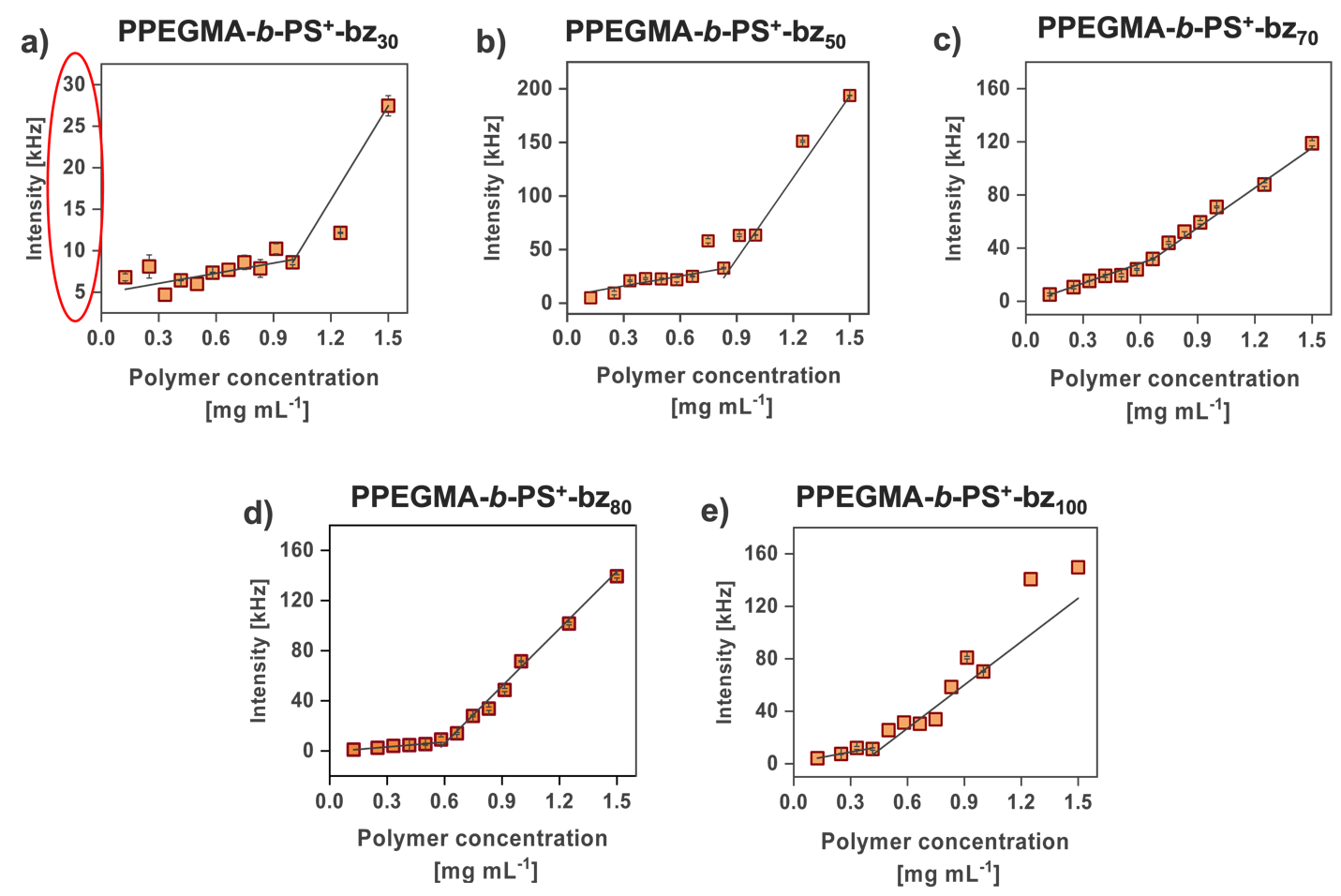


**Figure S19.** CMC analysis of sulfonium BCPs with penicillin G (1:1 molar ratio) via dynamic light scattering (DLS). Plots (a-e) depict scattering intensity [kHz] as a function of polymer concentration [mg mL^-1^], illustrating micelle formation at the x-intersection of both linear regions.

**
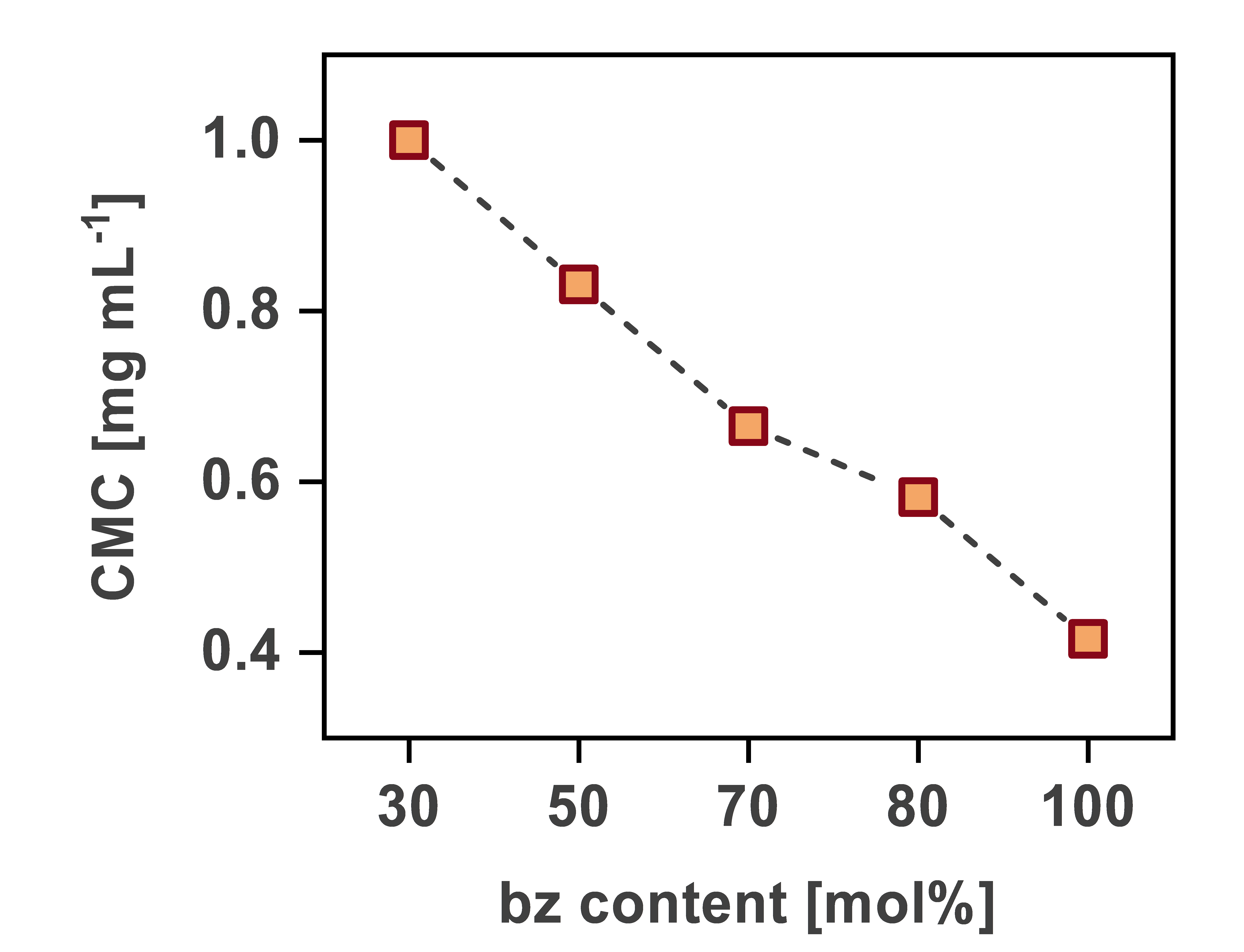
**

**Figure S20.** Critical micelle concentration (CMC) as a function of benzyl (bz) content [mol%]. Higher bz content correlates with lower CMC values, indicating more stable micelle formation, while lower bz content results in higher CMC, suggesting less stable or no micelles. This demonstrates the influence of bz content on micelle stability.

# **References**

[1] Gruber, Alexandra, Lucila Navarro, and Daniel Klinger. "Dual-reactive nanogels for orthogonal functionalization of hydrophilic shell and amphiphilic network." *Soft Matter* 18, no. 14 (2022): 2858-2871.

[2] Kanwal, Sidra, Umer Bin Abdul Aziz, Elisa Quaas, Katharina Achazi, and Daniel Klinger. "Sulfonium-based polymethacrylamides for antimicrobial use: influence of the structure and composition." *Biomaterials Science* 13, no. 4 (2025): 993-1009.

[3] Pham, Phuong, Susan Oliver, Duong Thanh Nguyen, and Cyrille Boyer. "Effect of cationic groups on the selectivity of ternary antimicrobial polymers." *Macromolecular Rapid Communications* 43, no. 21 (2022): 2200377.

[4] Oh, Junki, and Anzar Khan. "Main-chain polysulfonium salts: Development of non-ammonium antibacterial polymers similar in their activity to antibiotic drugs vancomycin and kanamycin." *Biomacromolecules* 22, no. 8 (2021): 3534-3542.

[5] Namivandi-Zangeneh, Rashin, Zahra Sadrearhami, Debarun Dutta, Mark Willcox, Edgar HH Wong, and Cyrille Boyer. "Synergy between synthetic antimicrobial polymer and antibiotics: a promising platform to combat multidrug-resistant bacteria." *ACS Infectious Diseases* 5, no. 8 (2019): 1357-1365.

[6] Aziz, Umer Bin Abdul, Ali Saoud, Marcel Bermudez, Maren Mieth, Amira Atef, Thomas Rudolf, Christoph Arkona et al. "Targeted small molecule inhibitors blocking the cytolytic effects of pneumolysin and homologous toxins." *Nature Communications* 15, no. 1 (2024): 3537.
